# Analysis of genetic and chemical variability of five *Curcuma* species based on DNA barcoding and HPLC fingerprints

**DOI:** 10.3389/fpls.2023.1229041

**Published:** 2023-09-06

**Authors:** Mengying Chen, Jian Sun, Hui Yao, Fuyu Gong, Long Cai, Chanyan Wang, Qingsong Shao, Zhian Wang

**Affiliations:** ^1^ Zhejiang Provincial Key Laboratory of Resources Protection and Innovation of Traditional Chinese Medicine, Zhejiang Agriculture and Forest University, Hangzhou, China; ^2^ Resource Center for Chinese Materia Medica, Zhejiang Research Institute of Traditional Chinese Medicine Co., Ltd., Hangzhou, China; ^3^ School of Pharmaceutical Sciences, Zhejiang Chinese Medical University, Hangzhou, China

**Keywords:** *Curcuma*, HPLC, DNA barcoding, ITS2, trnK

## Abstract

The rhizomes of *Curcuma* species have a long medicinal history in Asia. In China, *Curcuma* species mainly be utilized to make pharmaceutical products, including *C. phaecocaulis, C. aromatica, C. wenyujin, C. kwangsiensis* and *C. longa*. In this study, twenty-four samples were selected to study the genetic and chemical variability among five *Curcuma* species. The *ITS2* and *trnK* intron gene fragment were used to identify the five *Curcuma* species, the differences in chemical composition were computed using the Euclidean distance based on the data of HPLC characteristic peak areas and the content of six key components, and agronomic characteristics were analyzed including morphological and volatile oil characteristics. The *ITS2* and *trnK* intron gene fragment could distinguish the five *Curcuma* species clearly. The genetic distance between *Curcuma* species ranged from 0.0085 to 0.0767 based on the data of *ITS2* gene sequences with 32 variation sites, and the genetic distance between *Curcuma* species ranged from 0.0003 to 0.0194 based on the data of *trnK* intron gene sequences with 39 variation sites. Five *Curcuma* species showed otherness chemical composition characteristics, with the Euclidean distance ranging from 3.373 to 6.998. The *C. longa* showed the biggest variation compared with other species, with the Euclidean distance above 6.239. Among the samples of the original plants of Ezhu, the volatile oil yield of W1 was the highest, reached to 105.75 mL per single plant. Among all the samples, J6 showed the highest yield of volatile oil, reached to 149.42 mL per single plant. The results showed that chemical composition similarity of the medicinal plants was the primary proof for the selection of the original plants of the *Curcuma* medicinal materials. The genetic distance and chemical variability were important references for discovering new medicinal plant resources.

## 1 Introduction

There are many species of *Curcuma*, with at least 120 different species worldwide (Záveská et al., 2012). *Curcuma* species are widely distributed in 17 provinces of China, among which *C. longa* L., *C. aromatica* Salisb, *C. phaecocaulis* Val., *C. wenyujin* Y. H. Chen et *C.* Ling and *C. kwangsiensis* S. G. Lee et *C.* F. Liang are most commonly used (Zhang et al., 2018). The *Chinese Pharmacopoeia* records three Chinese medicine materials derived from *Curcuma* rhizomes including Jianghuang (*CURCUMAE LONGAE RHIZOMA*), Pianjianghuang (*WENYUJIN RHIZOMA CONCISUM*) and Ezhu (*CURCUMAE RHIZOMA*) with the effect of clearing veins and reducing pain. The original plants of Jianghuang and Pianjianghuang are *C. longa* and *C. wenyujin*, respectively. While, the original plants of Ezhu are *C. wenyujin*, *C. kwangsiensis*, and *C. phaecocaulis*. Volatile oil obtained from *Curcuma* species dried rhizomes have also shown pharmacological effects that include anti-cancer, anti-inflammatory and other properties (Guo et al., 2013; Zhang et al., 2017; Wu et al., 2021). The Ezhu You, which is the volatile oil obtained derived only from the rhizomes of *C. wenyujin*, is also recorded in *Chinese Pharmacopoeia*.

DNA barcoding is a method that can effectively identify species according to the short DNA fragments information. Ribosomal DNA (rDNA) including gene fragments such as *ITS1* and *ITS2* is commonly used to identify species. In the rDNA region, the internal transcribed spacer (ITS) as a high-mutant non-coding area provides more information sites in system development and distinguishes differences between *Curcuma* species (Pedersen, 2004). Compared to *ITS1*, *ITS2* has lower length variation and more common primer sites, which can better elucidate the genetic relationship between species (Tedersoo et al., 2015; Nilsson et al., 2019). The chloroplast DNA (cpDNA) including gene fragments such as *trnH-psdA*, *matK*, and *trnK* intron is commonly used to identify species (Minami et al., 2009; Záveská et al., 2012; Vinitha et al., 2014; Chen, 2015; Liu et al., 2022). Several researches have demonstrated that *trnK* intron gene segments are effective at identifying *Curcuma* species (Duan et al., 2017; Liu et al., 2022). Therefore, we chose the *trnK* intron and *ITS2* gene to evaluate the genetic distance between different *Curcuma species*.

It has been reported that the main components of the *Curcuma* species rhizomes are sesquiterpenes, such as bate-elemene, furanodienon, curdione, curzerene, germacrone and curcumenol (Xiang et al., 2012; Wang Y. et al., 2021). The chemical composition of both Ezhu (*CURCUMAE RHIZOMA*) and Jianghuang (*CURCUMAE LONGAE RHIZOMA*) are mainly terpenoids. Meanwhile the Jianghuang (*CURCUMAE LONGAE RHIZOMA*) specifically contain many curcuminoids, including curcumin, desmethoxycurcumin, and bisdemethoxycurcumin (Zhang et al., 2008; Ren et al., 2021; Wang et al., 2021b; Wang et al., 2021a). High-Performance Liquid Chromatography (HPLC) is a classical method to evaluate the content of chemical composition of Chinese herbs. And, more chemical information can be obtained from the HPLC fingerprints with clear and intuitive chromatography (Zhang et al., 2013).

In this study, *ITS2* and *trnK* intron gene sequences were used to analyze the genetic distance between different *Curcuma* species. To examine the chemical composition variation of the rhizomes of five *Curcuma* species, we established HPLC fingerprints and measured the quantity of 6 key components in the samples. And then, the relationship between genetic distance and chemical composition variation were also analyzed. Finally, major morphological and volatile oil characteristics were examined, which could provide a guide for *Curcuma* medicinal industry.

## 2 Materials and methods

### 2.1 Experimental materials

A total of 24 experimental materials had been collected from major producing areas in China, including 5 samples of *C. phaecocaulis*, 6 samples of *C. aromatica*, 4 samples of *C. wenyujin*, 3 samples of *C. kwangsiensis*, and 6 samples of *C. longa* (**Table 1**). The rhizomes of 22 samples (except G2 and G3) were harvested and dried in December in Hangzhou (Latitude: 119.97554°; Longitude: 30.36784°) with a growth period of one year. In addition, two fresh *C. kwangsiensis* samples (G2 and G3) were collected from Guangxi in June 2023 to study genetic distance, while the corresponding dry rhizomes also harvested and dried in December 2022 were collected to study the chemical composition.

**Table 1:** The agronomic traits of experimental samples (n = 3).

Specimen	Locality of collection	Latitude and longitude	Plant height / m	Leaf sheath color	Leaf villi		Leaf midrib characteristics	Rhizome color in inner section	Rhizome dry weight per single plant / g	Primary and secondary rhizome weight ratio	Volatile oil			Scientific name
					Up	Down					Production rate / (mL/g)	Yield per single plant / mL	Color	
P1	Xianyou County, Putian City, Fujian Province	25.36203°, 118.69183°	1.66±0.06	Brown	+	+	Purple-cored band	White endothelium with blue ring	564.94±21.77	3.62	0.18±0.02	98.86±5.98	Dark brown	*C. phaecocaulis*
P2	Huangpi Town, Xingning City, Meizhou City, Guangdong Province	24.13654°, 115.73119°	1.57±0.05	Brown	+	+	Purple-cored band	Blue endothelium with green ring (darker)	307.80±13.31	3.24	0.21±0.00	63.87±1.09	Light brown	*C. phaecocaulis*
P3	Gangnan District, Guigang City, Guangxi Zhuang Autonomous Region	23.11084°, 109.57339°	1.64±0.03	Brown	+	+	Purple-cored band	Blue endothelium with green ring (lighter)	332.43±54.55	3.20	0.21±0.01	68.15±2.35	Light brown	*C. phaecocaulis*
P4	Debao County, Baise City, Guangxi Zhuang Autonomous Region	23.90115°, 106.61897°	1.60±0.16	Brown	+	+	Purple-cored band	Blue endothelium with green ring (lighter)	195.06±23.85	2.05	0.26±0.02	51.20±3.45	Light brown	*C. phaecocaulis*
P5	Dagan Town, Shunchang County, Nanping City, Fujian Province	26.92980°, 117.74270°	1.25±0.11	Brown	+	+	Purple-cored band	Blue endothelium with green ring (darker)	120.01±41.58	1.80	0.15±0.00	18.30±0.42	Light brown	*C. phaecocaulis*
Y1	Sanjiang Town, Chongzhou City, Sichuan Province	30.54225°, 103.78474°	1.36±0.09	Green	–	+	Green	Yellow	248.43±31.34	0.48	0.41±0.01	100.61±1.76	Brownish yellow	*C.aromatica*
Y2	Nanping City, Guangxi Zhuang Autonomous Region	27.33174°, 118.12043°	1.36±0.05	Green	–	+	Green	Yellow	213.59±40.19	0.73	0.33±0.01	71.02±2.27	Brownish yellow	*C. aromatica*
Y3	Lingshan County, Qinzhou City, Guangxi Zhuang Autonomous Region	22.41650°, 109.29094°	1.36±0.06	Green	–	+	Green	Yellow	45.30±14.80	2.68	0.33±0.02	15.06±0.80	Brownish yellow	*C. aromatica*
Y4	Raoping County, Chaozhou City, Guangdong Province	23.66412°, 117.00390°	1.26±0.05	Green	–	+	Green	Yellow	229.97±18.52	0.65	0.37±0.01	84.86±2.93	Brownish yellow	*C. aromatica*
Y5	Wuguishan Town, Zhongshan City, Guangdong Province	22.44529°, 113.40282°	1.14±0.05	Green	–	+	Green	Yellow	166.49±31.38	0.77	0.30±0.00	49.53±0.59	Brownish yellow	*C. aromatica*
Y6	Dagan Town, Shunchang County, Nanping City, Fujian Province	26.92980°, 117.74267°	1.13±0.05	Green	–	+	Green	Yellow	108.92±5.46	0.55	0.27±0.00	29.62±0.31	Brownish yellow	*C. aromatica*
W1	Fengshukeng Village, Dongtou County, Yongjia City, Wenzhou City	27.96074°, 121.12156°	1.22±0.08	Green	–	–	Green	White endothelium with yellow ring	168.91±17.33	5.25	0.42±0.04	96.05±9.70	Dark purple	*C.wenyujin*
W2	Shazhou Village, Taoshan Town, Ruian City, Wenzhou City	27.82263°, 120.50856°	1.45±0.04	Green	–	–	Green	White endothelium with yellow ring	161.38±6.54	3.64	0.32±0.00	51.78±0.57	Dark purple	*C. wenyujin*
W3	Sanjia Village, outside Mayu Town, Ruian City, Wenzhou City	27.81026°, 120.45891°	1.47±0.05	Green	–	–	Green	White endothelium with yellow ring	239.29±49.75	5.13	0.40±0.01	59.28±1.06	Dark purple	*C. wenyujin*
W4	Dupu Village, Tonglingshan Town, Wencheng County, Wenzhou City	27.90992°,119.81282°	1.30±0.17	Green	–	–	Green	White endothelium with yellow ring	265.93±49.30	3.44	0.50±0.01	63.09±0.80	Dark purple	*C. wenyujin*
G1	Dagan Town, Shunchang County, Nanping City, Fujian Province	26.92980°, 117.74270°	0.63±0.05	Green	+	+	Green	White	317.24±56.75	2.11	0.20±0.01	63.45±4.85	Bright purple	*C. kwangsiensis*
G2	Zhenlong Town, PingnanCountry, Guigang City, Guangxi Zhuang Autonomous Region	23.41234°, 110.41226°	/	/	/	/	/	White	/	/	0.23±0.01	/	Bright purple	*C. kwangsiensis*
G3	Longsheng Town, Yulin City, Guangxi Zhuang Autonomous Region	22.53895°, 110.45475°	/	/	/	/	/	White	/	/	0.18±0.01	/	Bright purple	*C. kwangsiensis*
J1	Sanjiang Town, Chongzhou City, Sichuan Province	30.63543°, 103.69590°	1.55±0.11	Green	–	–	Green	Orange	228.69±1.46	0.65	0.41±0.01	108.20±3.73	Transparent light yellow	*C. longa*
J2	Jiujing Town, Qianwei County, Leshan City, Sichuan Province	29.07946°, 103.90546°	1.76±0.05	Green	–	–	Green	Orange	160.55±7.44	1.83	0.74±0.05	118.44±7.45	Transparent light yellow	*C. longa*
J3	Xilin County, Baise City, Guangxi Zhuang Autonomous Region	24.48957°, 105.09383°	0.84±0.22	Green	–	–	Green	Orange	150.06±29.82	0.96	0.58±0.00	97.55±0.60	Transparent light yellow	*C. longa*
J4	Longxu District, Wuzhou City, Guangxi Zhuang Autonomous Region	23.41513°, 111.24752°	1.47±0.04	Green	–	–	Green	Orange	125.05±18.99	1.27	0.46±0.01	73.43±1.14	Transparent light yellow	*C. longa*
J5	Shilong Town, Gaozhou City, Maoming City, Guangdong Province	22.04425°, 111.11426°	1.54±0.13	Green	–	–	Green	Orange	263.90±35.39	4.08	0.36±0.02	86.74±4.23	Transparent light yellow	*C. longa*
J6	Dagan Town, Shunchang County, Nanping City, Fujian Province	26.92980°, 117.74269°	0.96±0.10	Green	–	–	Green	Orange	159.62±38.37	0.69	0.55±0.01	146.79±2.63	Transparent light yellow	*C. longa*

Symbol (/) denotes not record; symbol (+) denotes observed; symbol (-) denotes not observed.

### 2.2 Chemical apparatus and instruments

The primers for *ITS2* and *trnK* sequencing were synthesized by Beijing Tsingke Biotechnology Co., Ltd. Six references including curdione, curcumenol, germacrone, curzerene, furanodienon, and beta-elemene were obtained from Chengdu nakeli-biotech Co., Ltd. M200 PRO Multimode Microplate Reader (Tecan, USA), Veriti™ 96-Well Fast Thermal Cycler (Thermo Fisher Scientific, USA), PowerPac™ Basic Power Supply (Bio-Rad, USA), and GelDoc™ XR+ Gel System (Bio-Rad, USA) for DNA extraction and PCR amplification. Agilent 1100 High-Performance Liquid Chromatography (Agilent, USA) with an Agilent ZORBAX Eclipse XDB-C18 Column (4.6mm×250mm, 5μm) for HPLC analysis.

### 2.3 DNA extraction, amplification, and sequencing

Total genomic DNA at the concentration of 100-150 ng was extracted from fresh leaves by the CTAB method. The extracted genomic DNA was amplified by polymerase chain reaction (PCR), using the *ITS2* (*ITS2ZF*, 5’-CTCTTGCATCATGAAGAACGT-3’ and *ITS8ZR*, 5’-TAGGGGAATCCTCGTAAGTTTC-3’) and *trnK* (*trnK3914F*, 5’-TGGGTTGCTAACTCAATGG-3’ and *CT828R*, 5’-TGAAGCAGAGGTAGAGGAAC-3’; *CT2240F*, 5’-TTGCAAAGATTAAGTTCGGG-3’ and *CT2675R*, 5’-TGGATAATATTTCCTTTTTT-3’) (Pedersen, 2004; Kita et al., 2016). Each 50 μL reaction mixture contained 25 μL 2X Taq PCR Master Mix, 5 μL Genomic DNA, 5 μL of each 10 μM primer and 10 μL ddH_2_O. The PCR conditions for amplification were 1 cycle at 94°C for 5 min; 35 cycles at 94°C 30 s, 56°C 30 s,72°C 50 s; and 1 cycle at 72°C for 10 min. The PCR products were detected in a 1% agarose gel and sent to Beijing Tsingke Biotechnology Co., Ltd. for sequencing. All the newly annotated samples were submitted to NCBI, and the accession numbers were shown in Table S7.

### 2.4 Morphological identifications of plant

The plant height, stem color, leaf sheath color, leaf epidermal hair on the front and back sides of the leaf, midrib characteristics, rhizome dry weight per single plant, the weight ratio of primary rhizome to secondary rhizome, rhizome inner section color and other agronomic characteristics were recorded.

### 2.5 Extraction of volatile oil

The volatile oil was extracted according to the 2020 edition of the *Chinese Pharmacopoeia* in the 2204 volatile oil determination method A. 10.00g of dried and ground herbs and 500 ml of water and zeolite were added to a 1000 ml round bottom flask connecting the volatile oil analyzer to the reflux condenser. Added water from the upper end of the condenser to fill the graduated part of the volatile oil tester, slowly heated it to boiling in the electric heating sleeve, and kept it slightly boiling for 5 hours, until the amount of oil in the tester no more increase and stopped heating. When the temperature dropped to room temperature, read the volatile oil volume, and calculated the volatile oil content in the test solutions.

### 2.6 HPLC analysis

The test solutions were made by ultrasonically extracting 0.5 g of dried and ground herbs in 10 mL of methanol at room temperature for 30 minutes. The extracted solution was cooled, then added to the initial weight along with methanol. As for the standard solution, curdione (6.50 mg), curcumenol (5.04 mg), germacrone (4.14 mg), curzerene (7.58 mg), furanodienon (6.74 mg) and beta-elemene (12.03 mg) were respectively placed in a 10 mL volumetric flask and dissolved with methanol as stock solution. All test and standard solutions were filtered through a 0.45 μm filter before being used for HPLC analysis.

For HPLC analysis, 0.02% phosphoric acid aqueous solution (A) and acetonitrile (B) were used as the mobile phase in gradient elution mode. The elution gradient was set as follows: 0 - 20 min, 50% (B); 20 - 30 min, 50% - 60% (B); 30 - 42 min, 60% - 73% (B); 42 - 45 min, 73% - 88% (B); 45 - 50 min, 88% - 73% (B); 50 - 55 min, 73% - 85% (B); 55 - 65 min, 85% (B); 65 - 70 min, 85% - 50% (B); 70 - 80 min, 50% (B). The detection wavelength was 215 nm, the injection volume was 10 μL, the flow rate was 1.0 mL/min, and the column temperature was 30°C.

### 2.7 Data analysis

ContigExpress software was used to splice the two-way sequencing peak map and delete the weak or overlapping peak regions at both ends in order to obtain the DNA sequence. From GenBank, we retrieved and downloaded standard sequences for *ITS2* and *trnK* intron regions with high confidence that matched the sample sequences, containing five *Curcuma* species and an *Alpinia officinarum* Hance of the ginger family as external standard. There were 11 *ITS2* standard sequences. The sequence JQ409962.1 was *C. aromatica*; MF589398.1, MF589400.1, MF589399.1 and MF589397.1 were *C. wenyujin*; MF096202.1, MF589420.1, MF096205.1 and MF096201.1 were *C. phaeocaulis*; JQ409956.1 was *C. longa*; KY454008.1 was *C. kwangsiensis*; AF478718.1 was *Alpinia officinarum* Hance. There were 6 *trnK* intron standard sequences. AB047731.1 was *C. aromatica*; AB047746.1 was *C. wenyujin*; LC636646.1 was *C. longa*; LC636647.1 was *C. kwangsiensis*; AB047735.1 was *C. phaeocaulis*; AF478818.1 was *Alpinia officinarum* Hance. Using MEGA 7.14 software, the standard sequences and the sample sequences based on standard parameters to construct a Neighbour-joining tree with bootstrap testing of 1000 replicates and calculated the average intra-specific and inter-specific p-distance as the genetic distance.

The reference chromatogram was generated using a Similarity Evaluation System for Chromatographic Fingerprint of TCM (Version 2012). Origin Pro 2022 software was used to perform Principal component analysis (PCA) and hierarchical clustering analysis (HCA). HCA was applied using the Heat-mapper plug-in, which used the intergroup join method, and the distance formula for sample similarity was the square Euclidean distance. PCA used the Principal Component Analysis plug-in for unsupervised pattern recognition. IBM SPSS Stastics26.0 software was used to standardize the data of HPLC 17 characteristic peak areas and 6 key components contents of 24 samples, and the Euclidean distance between different *Curcuma* species was calculated using the standardized data. The Spearman correlation analysis results of the content of 6 key components in the five *Curcuma* species were obtained using IBM SPSS Stastics26.0 software, and the correlation analysis diagram was generated by the website https://www.chiplot.online/. One-way ANOVA was also performed using IBM SPSS Stastics26.0 software on the sample volatile oil production rate and yield (Production rate = Volatile oil weight (mL)/Powder weight (g), Yield = Production rate * Rhizome dry weight per single plant (g)).

We combined the *ITS2* and *trnK* intron sequences and constructed a p-distance matrix, designated Matrix A. Matrix B and Matrix C, correspondingly, were made using the Euclidean distance matrix by the data of standardizing the 17 characteristic peak areas and 6 chemical component contents of the HPLC between samples (**Table S4**, **Table S5** and **Table S6**). A 999 permutation Pearson correlation coefficient calculation was performed on the p-distance matrix and the Euclidean distance matrix using the vegan Mantel function in R to assess how well the chemical composition matched the genetic background.

## 3 Results

### 3.1 Agronomic characteristics of *Curcuma* species

**Figure 1:**
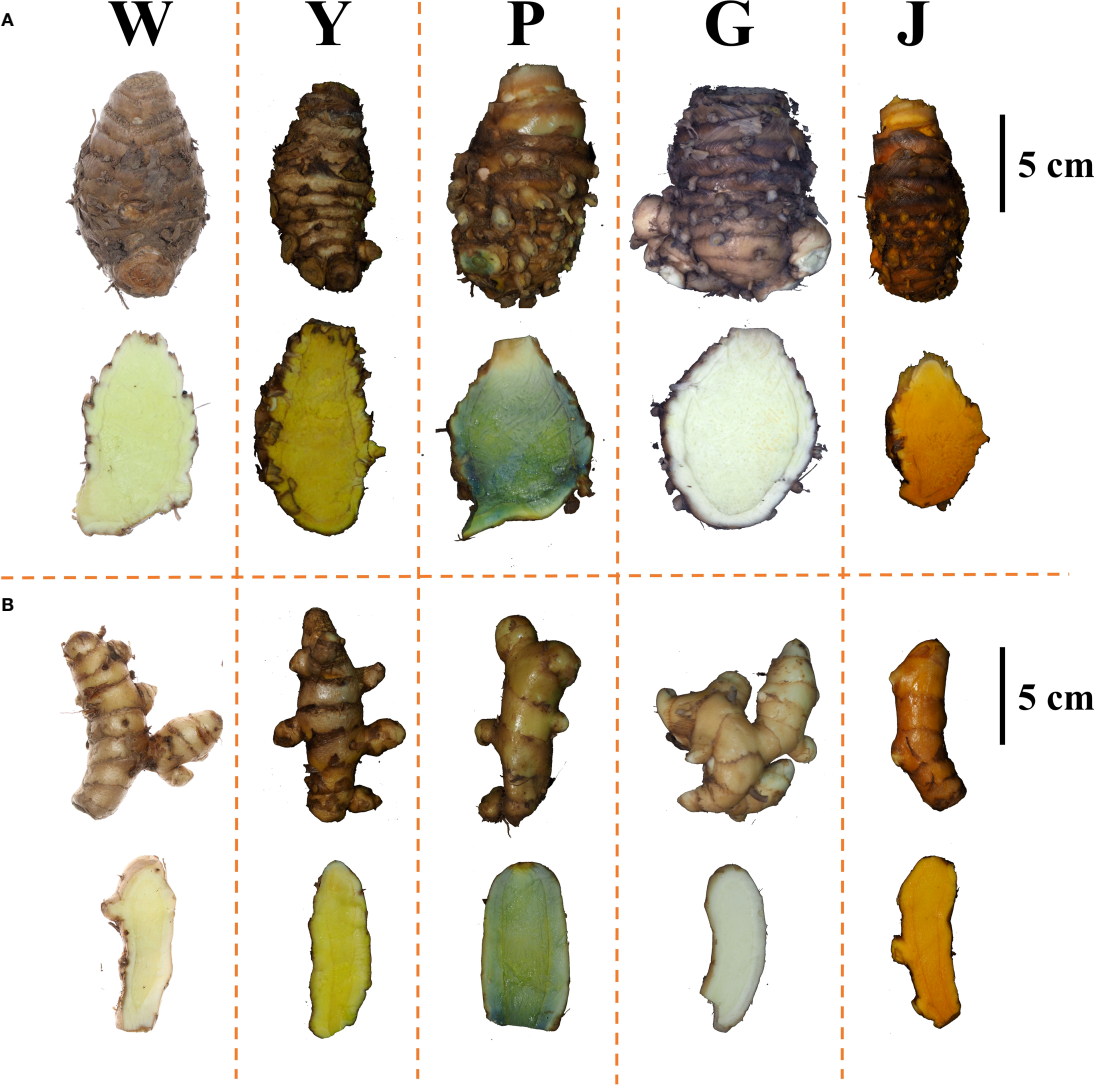
Rhizome morphology and inner section of five *Curcuma* species. **(A)** Morphological of the primary rhizomes; **(B)** Morphological of the secondary rhizomes; (W) *C. wenyujin*; (Y) *C. aromatica*; (P) *C. phaecocaulis*; (G) *C. kwangsiensis*; (J) *C. longa*.

In the germplasm resource garden, the samples were morphologically identified based on the characteristics of the *Curcuma* species above ground (plant height, color of the leaf sheath, presence or absence of epidermal hair on the leaf’s front and back surfaces, and characteristics of the leaf midrib) and underground (Rhizome dry weight per single plant, weight ratio of primary rhizome to secondary rhizome, and color of the rhizome inner section). The biomass of *C. phaecocaulis* and *C. kwangsiensis* was higher than the other species. The secondary rhizomes of *C. longa* were more developed, while the primary rhizomes were smaller and less developed. *C. wenyujin* and *C. aromatica*were similar in shape, with the primary distinctions being that the rhizome profile of the latter had a darker yellow hue and the back of the leaf was smooth and hairless as shown in **Table 1** and **Figure 1**. The morphological identification of five *Curcuma* species in this study were consistent with previous reports (Kita et al., 2016).

**Figure 2:**
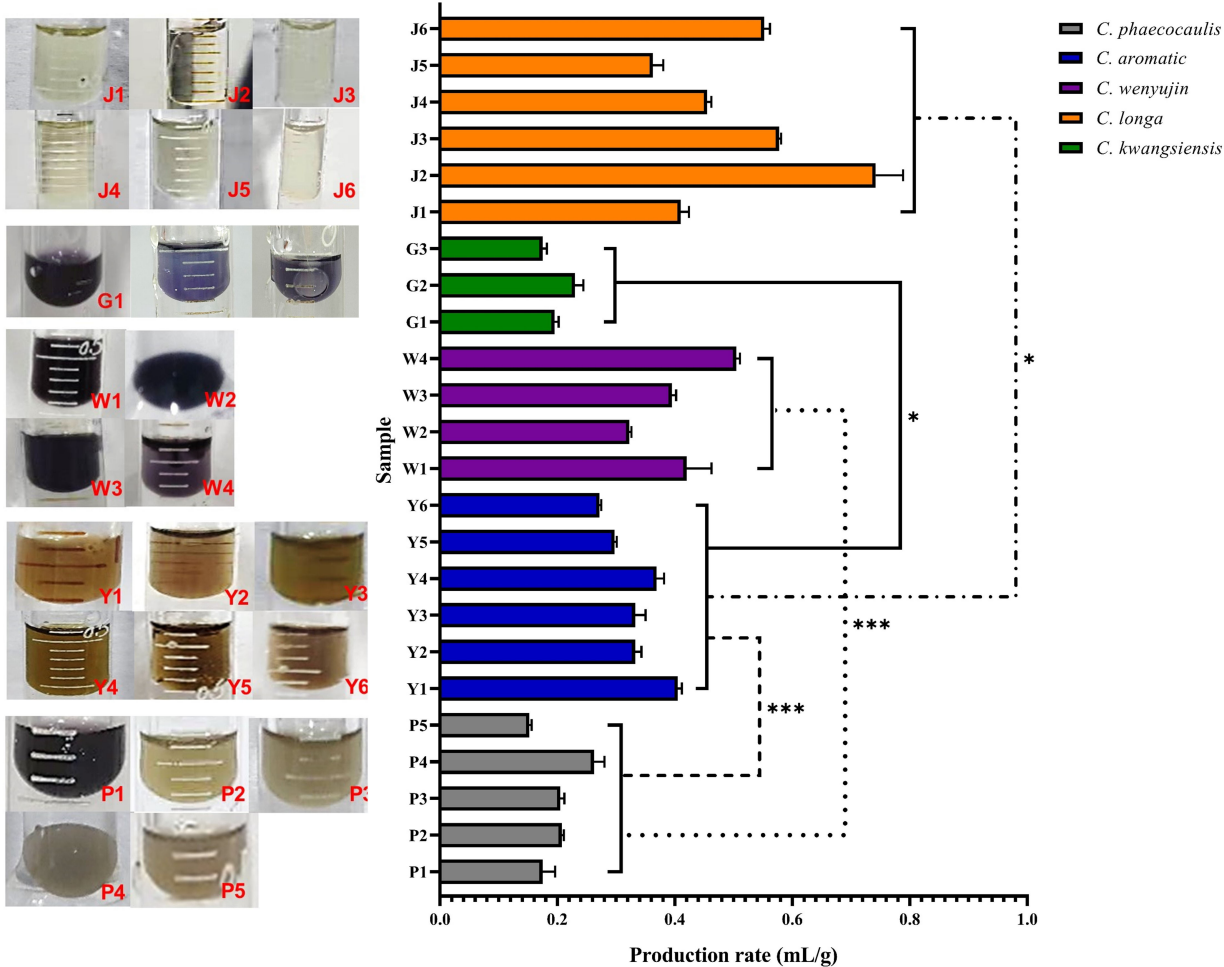
The volatile oil characteristics of five *Curcuma* Species (n = 3). Symbol (*) denotes P < 0.05; Symbol (***) denotes P < 0.001.

In the production and application of *Curcuma* medicinal materials, the yield and production rate of volatile oil were important productivity indicators, and color was the most rapid and direct important index for quality evaluation. The *Chinese Pharmacopoeia* also includes the extract of Ezhu You (*ZEDOARY TURMERIC OIL*), which is the volatile oil derived from the rhizomes of *C. wenyujin*. The volatile oil production rate of the dried rhizomes of the five *Curcuma* species was, from high to low: *C. longa* > *C. wenyujin* > *C. aromatica* > *C. kwangsiensis* > *C. phaecocaulis*. Among the samples of the original plants of Ezhu, the volatile oil yield of W1 was the highest, reached to 105.75 mL per single plant. Among all the samples, J6 showed the highest yield of volatile oil, reached to 149.42 mL per single plant (**Figure 2**, **Figure S1**). Meanwhile, the volatile oil derived from different species showing distinguishable colors, which were translucent light yellow, dark purple, brownish yellow, bright purple, and light brown, respectively. A significant difference in volatile oil color, production rate and yield among five *Curcuma* species which planted in Hangzhou. It revealed that W1 may the best variety to achieve the best economic benefits of Ezhu You in Hangzhou.

### 3.2 DNA barcode result analysis

#### 3.2.1 *ITS2* gene sequences

**Figure 3:**
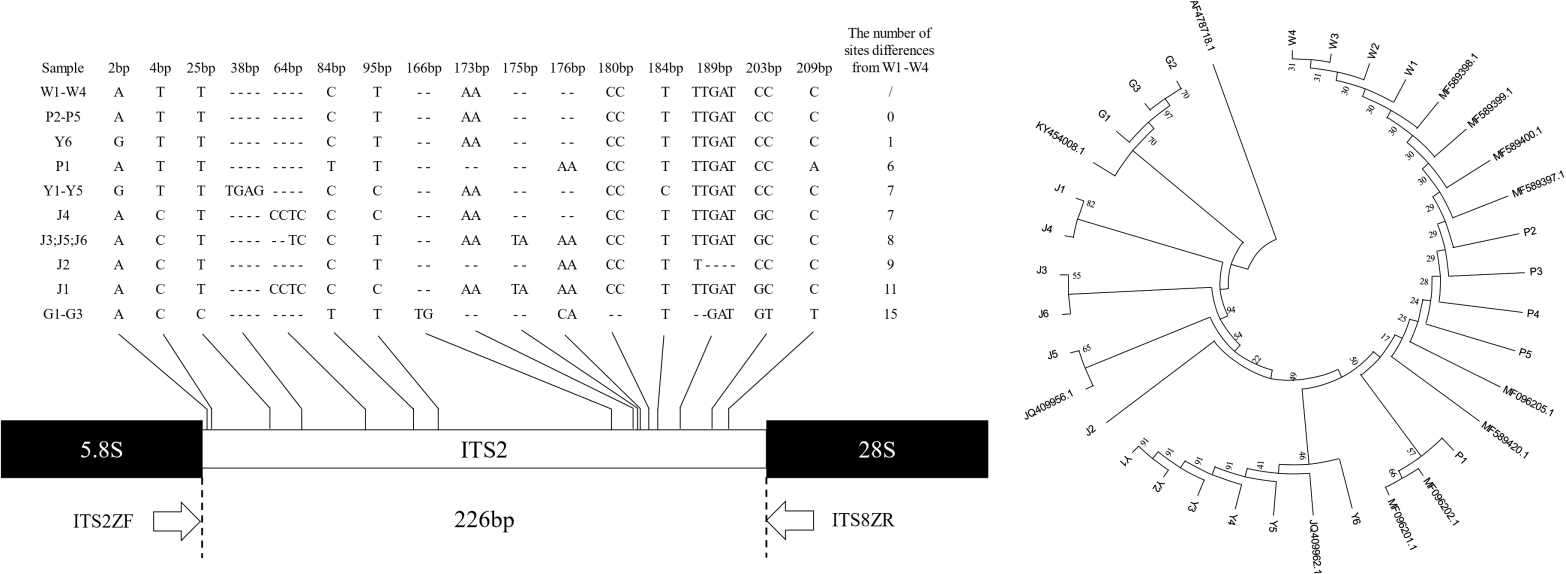
*ITS2* gene sequences and genetic relationship among five *Curcuma* species. Hyphens (-) denote alignment gaps.

PCR amplification of *ITS2* fragments of all samples using primers *ITS2ZF*/*ITS8ZR* was performed and successfully sequenced, resulting in 24 high-quality *ITS2* fragment sequences. The *ITS2* sequences were between 225 and 239 base pairs compared with the standard sequences (**Figure 3**); the shortest G1 - G3 sequences were 225 base pairs and the longest J1 sequence was 239 base pairs. The differences in *ITS2* region loci in the samples were shown in 0 - 32 differential loci (including 7 singleton loci and 9 continuous loci). When *C. wenyujin* samples W1 - W4 were set as reference sequences, the number of differential sites in *C. phaeocaulis*, *C. aromatica, C. longa*, and *C. kwangsiensis* was 0 - 6, 1 - 7, 7 - 11 and 15, respectively. There was no difference in *ITS2* region between samples P2 - P5 and W1 - W4. Fewer differences existed between Y1 - Y6 and W1 - W4, and G1 - G3 differed from W1 - W4 significantly.

We constructed a Neighbour-joining tree based on the *ITS2* gene fragment of 24 sample sequences and 11 standard sequences to examine the genetic distance between samples (**Figure 3**). The findings indicated that *Alpinia officinarum* Hance as an external standard, its genetic distance from samples was considerably greater. G1 - G3, J1 - J6, and Y1 - Y6 were each clustered into one unit and were identified as *C. kwangsiensis*, *C. longa* and *C. aromatica*. W1 - W4 and P1 - P5 were grouped together and identified as *C. wenyujin* and *C. phaeocaulis*. The intraspecific genetic distance ranged from 0.000 to 0.0230, the interspecific genetic distance ranged from 0.0085 to 0.0767, and the average distance was 0.03257. It was clarified that while the *ITS2* contained enough genetic variation to discriminate *Curcuma* species, but it was insufficient to distinguish between *C. wenyujin* and *C. phaeocaulis.*

#### 3.2.2 *TrnK* intron gene sequences

**Figure 4:**
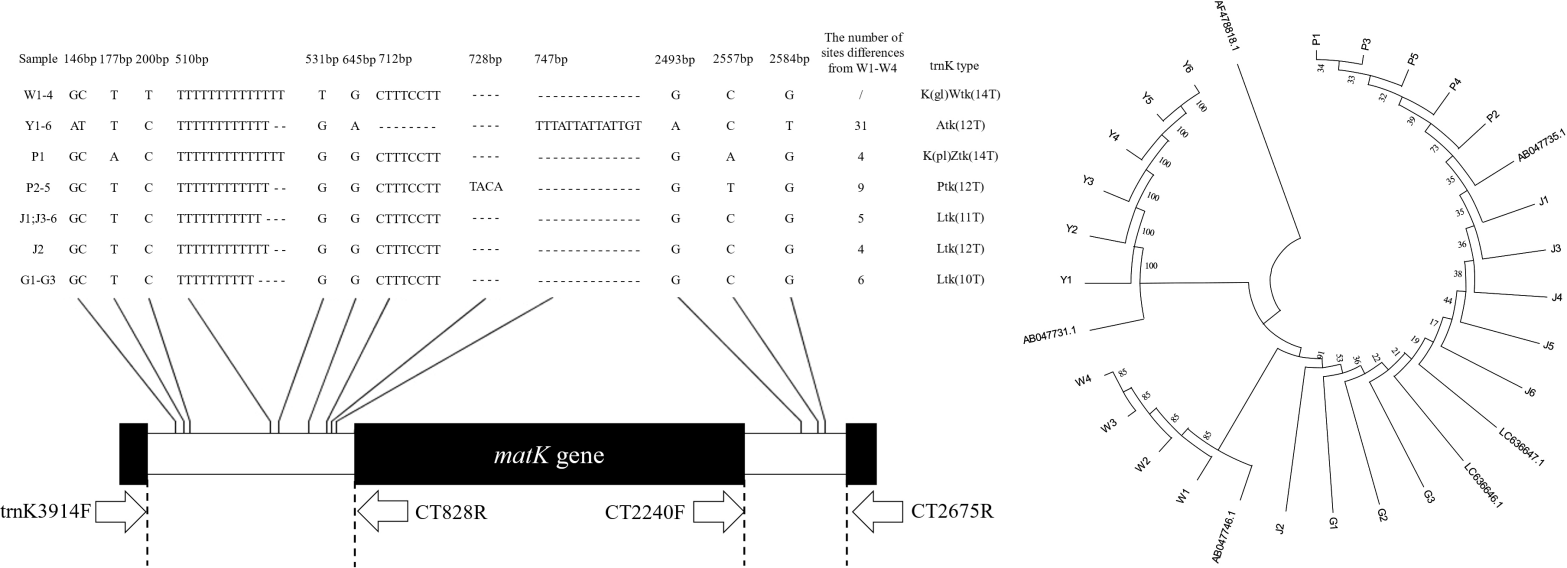
*TrnK* intron gene sequences and genetic relationship among five *Curcuma* species. Hyphens (-) denote alignment gaps.

According to the explanation of the *Curcuma* species *trnK* intron gene structure in the literature (Cao et al., 2002; Komatsu et al., 2008; Kita et al., 2016), the sample sequences were divided into 5 categories (K (gl) Wtk, Atk, K (pl) Ztk, Ptk, Ltk), and there were 10 - 14 continuous unequal thymine at the 501 loci (**Figure 4**). For the first time, we have found the Ltk (12T) structure in *C. longa* (J2), and the K (pl) Ztk (14T) structure in *C. phaeocaulis* (P1). *C. aromatica* had one or two base substitutions at 146, 645, 2493 and 2584, an 8 bp fragment deletion at 712 and a 14 bp fragment insertion at 747. A 4 bp gene fragment insertion at 728 in samples P2-P5. The sequence alignment results showed that the *trnK* intron gene structure regions of the five *Curcuma* species were highly conserved and only single base substitution and small fragment deletion or insertion existed among different samples.

The Neighbour-joining tree was constructed with the sample sequences and the standard sequences (**Figure 4**). The average genetic distance was 0.0078, the intraspecific genetic distance ranged from 0.0000 to 0.0028 and the interspecific genetic distance ranged from 0.0003 to 0.0194. C*. aromatica* and *C. wenyujin* were divided into separate branches, whereas *C. phaeocaulis, C. kwangsiensis*, and *C. longa* were placed together.

### 3.3 HPLC analysis

#### 3.3.1 HPLC fingerprints

**Figure 5:**
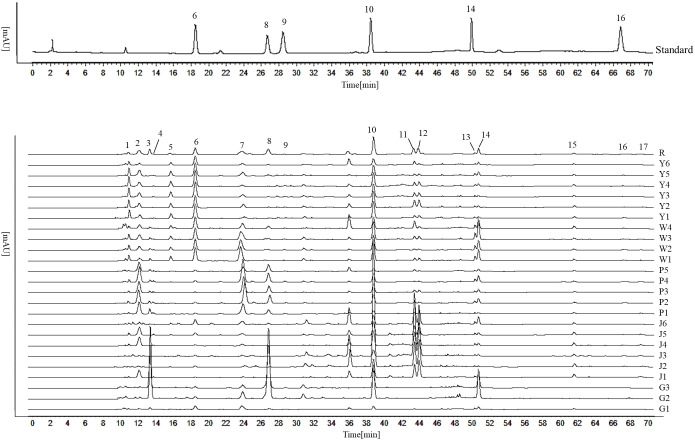
HPLC fingerprints of five *Curcuma* species. Peak 6 is Curdione; peak 8 is furanodienon; peak 9 is curcumenol; peak 10 is germacrone; peak 14 is curzerene; peak 16 is beta-elemene.

**Figure 6:**
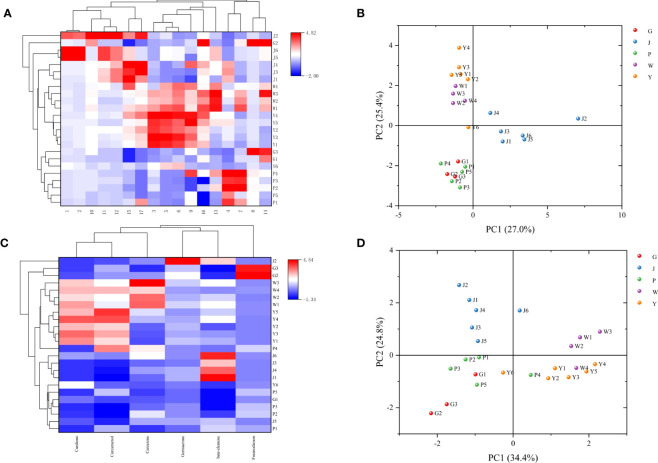
HCA and PCA analysis based on chemical composition. **(A)** HCA analysis based on the data of 17 characteristic peak areas; **(B)** PCA score based on the data of 17 characteristic peak areas; **(C)** HCA analysis based on the data of 6 chemicals; **(D)** PCA score based on the data of 6 chemicals.

The five *Curcuma* species could be distinguished using the HPLC fingerprints, however, each sample could not be distinguished with absolute precision (**Figure 5**). We attempted to identify five *Curcuma* species using HPLC and examined the chemical variations among them. The results of HCA and PCA revealed that the chemical composition of five *Curcuma* species rhizomes were different (**Figure 6**). The HPLC fingerprints of *C. wenyujin, C. aromatica, C. phaeocaulis, C. kwangsiensis* and *C. longa* showed 10, 6, 3, 3 and 8 distinctive peaks with larger peak areas, respectively. The HPLC fingerprints among the samples were quite different, and the similarity ranged from 0.211 to 0.999. The differences among samples of the same species were relatively small, and the similarity was greater than 0.599 (**Table S1**). In order to understand species with similar chemical composition, we used 17 characteristic peak areas of the HPLC fingerprints to analyze the Euclidean distance of five *Curcuma* species (**Table S2**), including *C. wenyujin* and *C. aromatica* (3.373)at distance less than 5, *C. phaeocaulis* and *C. kwangsiensis* (5.209), *C. wenyujin* and *C. phaeocaulis* (5.322), *C. wenyujin* and *C. kwangsiensis* (5.361), *C. aromatica* and *C. phaeocaulis* (5.752), *C. aromatica* and *C. kwangsiensis* (5.960) at distance ranged from 5 to 6, and distance greater than 6 were *C. longa* and *C. phaeocaulis* (6.861), *C. longa* and *C. wenyujin* (6.239), *C. longa* and *C. aromatica* (6.392), *C. longa* and *C. kwangsiensis* (6.998). The findings revealed differences in chemical composition between *C. longa* and the other four species. The results also explained the reason why the *Chinese Pharmacopoeia* defines *C. wenyujin*, *C. phaeocaulis* and *C. kwangsiensis* as the original plants of Ezhu and *C. longa* as the original plant of the Jianghuang. It also demonstrated that *C. aromatica* has the capacity to be developed into one of the original plants of Ezhu.

#### 3.3.2 Chemical composition analysis

**Figure 7:**
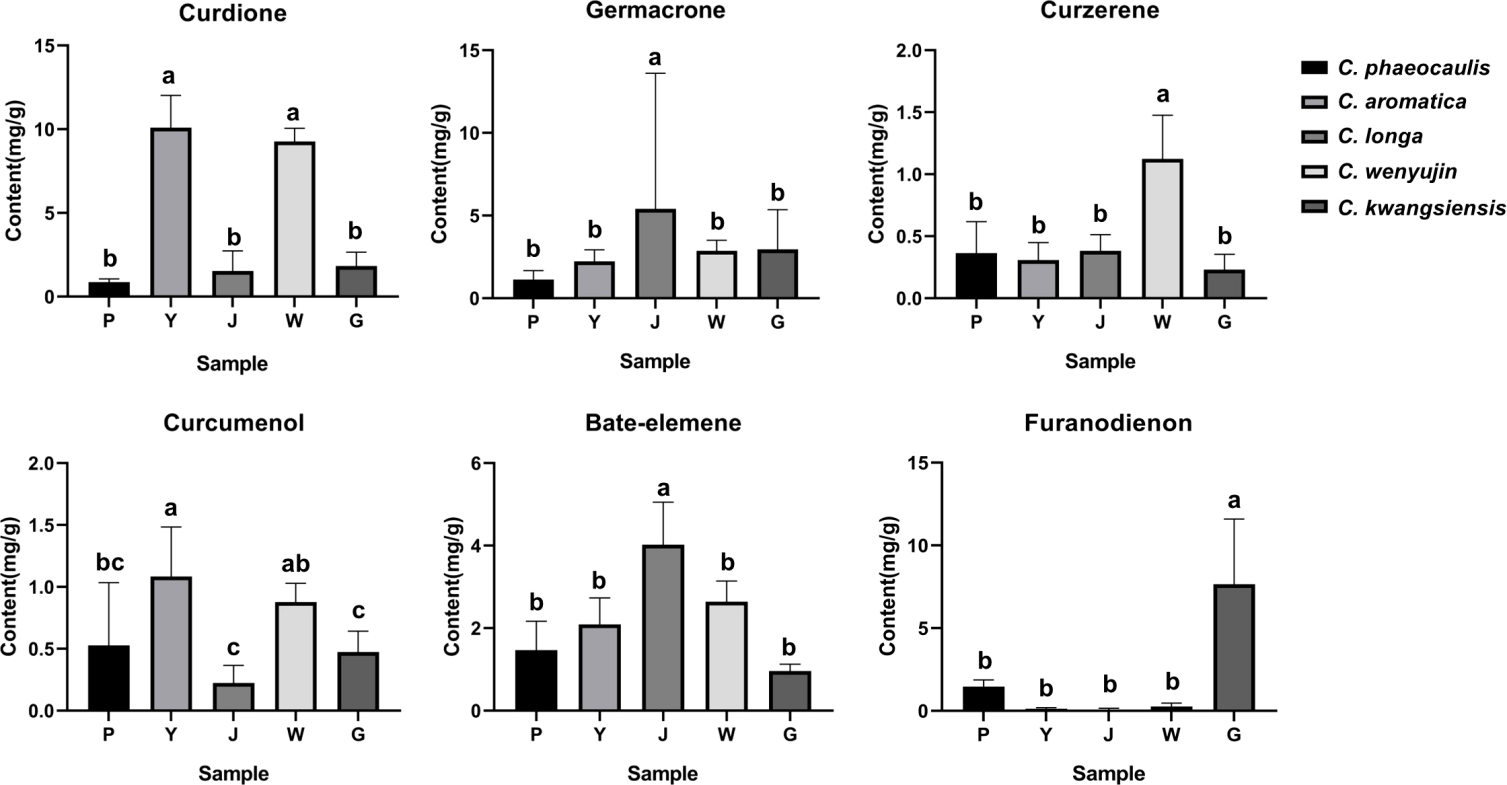
Comparison chart of 6 key components content of five *Curcuma* species. Different lowercase letters represent significant differences.

We quantitatively examined six chemicals in the samples: curdione, curcumenol, germacrone, curzerene, furanodienon and beta-elemene, in order to further investigate the material basis that resulted in the distinct chemical composition across *Curcuma* species (**Table S3**, **Figure 7**). The content of curzerene (0.7883 - 1.6192 mg/g) and curdione (8.4492 - 10.2745 mg/g) were higher in *C. wenyujin*. The content of curdione (6.8979 - 12.1390 mg/g) and curcumenol (1.0091 - 1.4891 mg/g) were higher in *C. aromatica*. In *C. phaeocaulis*, there was a lot of furanodienon (0.8945 - 1.9348 mg/g), and beta-elemene was abundant in *C. longa* (2.4254 - 5.1776 mg/g). HCA and PCA results revealed that although different *Curcuma* species contained high levels of the six chemicals, it was challenging to distinguish among them on these results (**Figure 6**).

#### 3.3.3 Metabolic pathways of terpenoids

**Figure 8:**
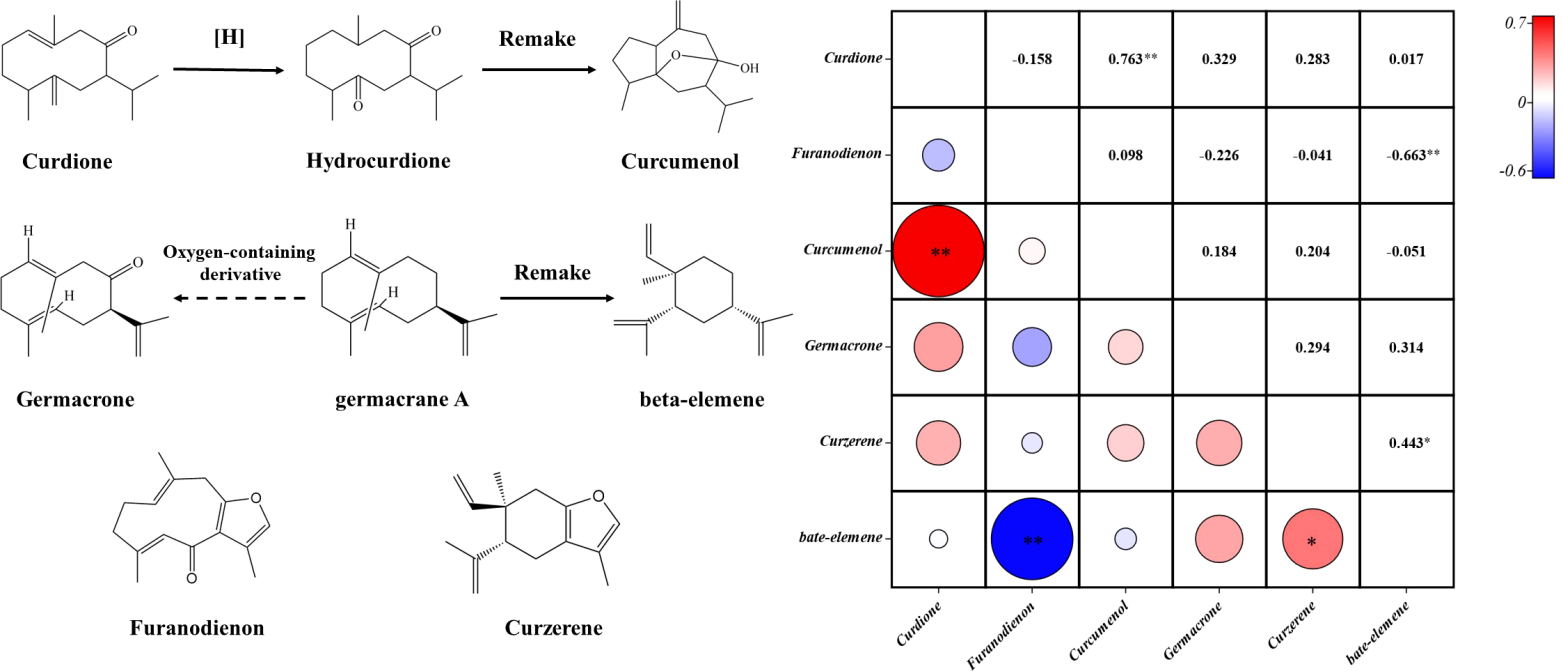
Diagrams of the relationship among the six components of the five *Curcuma* species. Symbol (*) denotes P < 0.05; Symbol (**) denotes P<0.01.

The Spearman correlation analysis was performed on six components. Components with a significant positive correlation were curdione and curcumenol, curzerene and beta-elemene. Furanodienon had a significant negative correlation with beta-elemene. The content of germacrone and beta-elemene was positively correlated, and the similarity coefficient of was 0.314 (**Figure 8**). Under certain conditions, curdione can be converted into curcumenol (Shiobara et al., 1985), germacrone is an upstream compound of beta-elemene synthesis (Barrero et al., 2011), which explained the correlation between curdione and curcumenol, germacrone and beta-elemene (**Figure 8**). But there was little evidence to support a relationship between the biogenic production processes of other components. Our findings suggested that among the *Curcuma* species, curzerene and beta-elemene might be upstream and downstream products in the synthetic pathway. Furanodienon might compete the same upstream product in the synthetic pathway with beta-elemene.

### 3.4 Relationship between genetic distance and chemical variation

The Mantel test is a method for comparing sample distances in two sets of distance measurement matrices. In the current study, we employed the Mantel test to investigate the relationship between 24 samples’ chemical composition and the degree of genetic variation (Duan et al., 2017).The Mantel test function based on Pearson’s coefficient in R was used to test the correspondence between the P-distance matrix and the Euclidean distance matrix. The results showed that matrix A and matrix B were weak positive correlation (r = 0.2994, p = 0.004), and matrix A and matrix C were also weak positive correlation (r = 0.2789, p = 0.005), indicating that there was a weak correspondence between the genetic and chemical variability (Duan et al., 2017).

## 4 Discussion

### 4.1 Analysis of genetic distance between *Curcuma* species

Our survey showed a number of *Curcuma* species were utilized in the manufacture of medicines in China. The four different species of *Curcuma* listed in the *Chinese Pharmacopoeia* are *C. wenyujin*, *C. longa, C. kwangsiensis*, and *C. phaeocaulis*. *C. wenyujin* is a variety of *C. aromatica* Salisb found in *Flora of China*, and *C. aromatica* Salisb is also used in large quantities in production, so we included it as one of the research objects.

Several medicinal materials recorded in *Chinese Pharmacopoeia* originated from different species of plants. Such as Huangjing (*POLYGONATI RHIZOMA*) originated from three species including *Polygonatum kingianum* Coll. et Hemsl., *Polygonatum sibiricum* Red and *Polygonatum cyrtonema* Hua. Studies have revealed that the genetic gap between Huangjing original plants is much closer than that of other Polygonatum species (Park et al., 2017; Feng et al., 2020). In this study, we found that *ITS2* and *trnK* intron gene fragment could distinguish the five *Curcuma* species very well. Similar to previous studies, C. longa showed a relatively farther genetic distance from other Curcuma species, including C. wenyujin, C. aromatica, C. kwangsiensis, and C. phaecocaulis showed closer genetic distance to each other (Kita et al., 2016; Deng et al., 2018). However, the correlation between genetic distance based on finite gene sequence and chemical variability showed a relatively low level. As finite sequence of several genes cannot reflect the genetic distance, and may not precisely predict the relationship between genetic distance and chemical variation. Then, more molecular markers whole genome information can be applied to analyze the genetic distance between *Curcuma* species to furtherly explore the correlation between genetic and chemical variability. The potential pharmacodynamics of new species can be predicted by analyzing the genetic distance between species of the same genus and known medicinal pants.

### 4.2 Differential effect based on chemical composition variation of different *Curcuma* medicinal materials

Numerous bioactive ingredients in herbs are the material basis of pharmacological effects. However, it is a very difficult task to evaluate the efficacy of medicinal herbs, due to the complex chemical composition and the interaction among components. For instance, furanodienon, beta-elemene, curdione, and germacrone can all act on many cancer cell targets and have positive anti-cancer effects (Zeng JH. et al., 2012; Zhong et al., 2014; Huang et al., 2017), and breast cancer cell proliferation is significantly reduced when curdione, germacrone, and furanodienon are combined (Kong et al., 2013). Modern pharmacological studies have found that *Curcuma* rhizomes extract has effective anti-cancer properties. However, different *Curcuma* species have different targets in different cancer models. For example, *C. wenyujin* can increase apoptosis in hepatoma cells by inducing increased expression of apoptotic genes Bid and Bax, and decreased expression of anti-apoptotic gene Bcl2 (Liu et al., 2019). Similarly, *C. kwangsiensis* can induce apoptosis of nasopharyngeal cancer cells by reducing the expression of Bcl2 and promoting the expression of p53 (Zeng J. et al., 2012). Furthermore, *C. phaeocaulis* can inhibit the growth of liver cancer cells by inhibiting STAT3 activity (Dong et al., 2018), and *C. longa* acts as a PARP inhibitor to induce apoptosis in cervical cancer cells (Li et al., 2014) and so on. According to the results of our current studies, the chemical composition of *C. wenyujin* and *C. aromatica*, *C. phaeocaulis* and *C. kwangsiensis* was the most similar, and the Euclidean distance was 3.373 and 5.209, respectively. The chemical composition between *C. longa* and others were the largest, with the Euclidean distance were above 6.239. Therefore, we hypothesized that the components was the main factor in the selection of the original plants of the medicinal material, and thus they shared similar pharmacological effects.

## 5 Conclusion

The original plants of medicinal material recorded in the *Chinese pharmacopoeia* has been clearly specified, which is the primary proof of safety and effectiveness in application. Only a limited number of medicinal plants have been recorded in the *Chinese pharmacopoeia*, but there are many medicinal plants of the same genus that have not been fully studied. To determine whether a plant has the potential similar efficacy as a known herb is the first problem to be solved for discovering new medicinal plant resources. According to this research, the genetic distance data could provide some reference clues to find new medicinal plant resources. While the basic basis of different original plants for Jianghuang and Ezhu, both derived from *Curcuma* species, is mainly according to the differences of chemical composition. As the Euclidean distance of the chemical composition of *C. aromatica* and *C. wenyujin* is 3.373, which is much lower than that between *C. wenyujin* and *C. phaeocaulis* reached to 5.332. Therefore, *C. aromatica* has the potential to be the original plant for Ezhu.

## Data availability statement

The original contributions presented in the study are included in the article/**Supplementary Material**, further inquiries can be directed to the corresponding author.

## Author contributions

ZW, JS and MC designed the experiment. MC, FG, LC and CW performed the experiment. MC and HY participated in the data analysis and prepared the manuscript. ZW, JS and QS revised the manuscript. All authors read and approved the final article.
