# Supplementary figures and images for: Analysis of genetic and chemical variability of five Curcuma species based on DNA barcoding and HPLC fingerprints
Source: Front Plant Sci. 2023 Sep 6;14:1229041. doi: 10.3389/fpls.2023.1229041 (PMC10511903; doi:10.3389/fpls.2023.1229041)

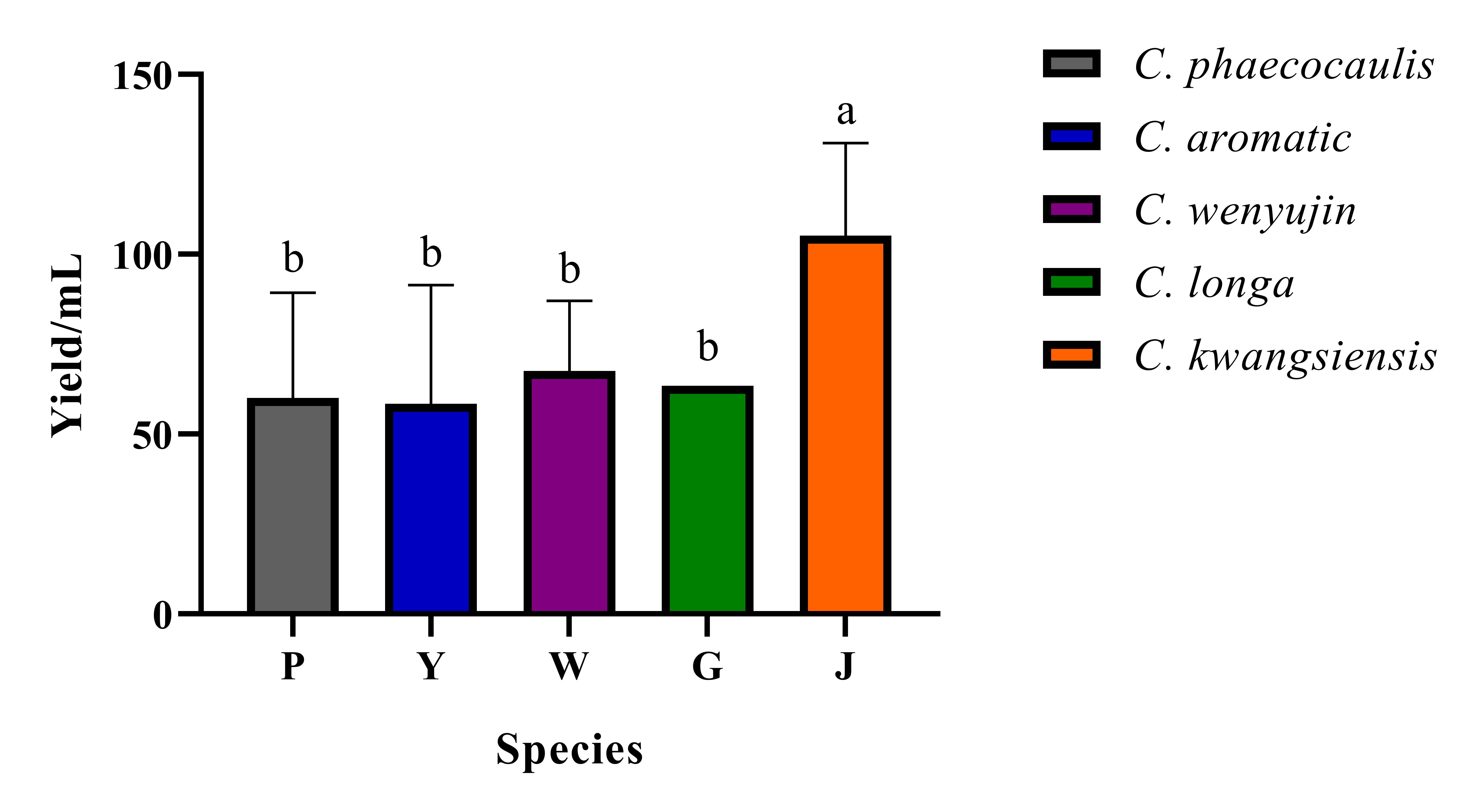

Supplement: Supplementary file 1 [file Image_1.jpeg]
