# Supplementary material for: Analysis of genetic and chemical variability of five Curcuma species based on DNA barcoding and HPLC fingerprints
Source: Front Plant Sci. 2023 Sep 6;14:1229041. doi: 10.3389/fpls.2023.1229041 (PMC10511903; doi:10.3389/fpls.2023.1229041)
Supplement: Supplementary file 2 [file Table_1.docx]

Table S1 HPLC fingerprints similarity

| Sample | G1 | G2 | G3 | J1 | J2 | J3 | J4 | J5 | J6 | P1 | P2 | P3 | P4 | P5 | W1 | W2 | W3 | W4 | Y1 | Y2 | Y3 | Y4 | Y5 | Y6 | R |
| --- | --- | --- | --- | --- | --- | --- | --- | --- | --- | --- | --- | --- | --- | --- | --- | --- | --- | --- | --- | --- | --- | --- | --- | --- | --- |
| G1 | 1.000 | 0.604 | 0.599 | 0.449 | 0.386 | 0.704 | 0.555 | 0.613 | 0.892 | 0.845 | 0.857 | 0.865 | 0.858 | 0.940 | 0.954 | 0.966 | 0.925 | 0.902 | 0.710 | 0.773 | 0.767 | 0.712 | 0.844 | 0.792 | 0.822 |
| G2 | 0.604 | 1.000 | 0.999 | 0.979 | 0.962 | 0.985 | 0.996 | 0.991 | 0.873 | 0.466 | 0.548 | 0.631 | 0.537 | 0.671 | 0.807 | 0.781 | 0.840 | 0.733 | 0.758 | 0.813 | 0.662 | 0.661 | 0.681 | 0.595 | 0.942 |
| G3 | 0.599 | 0.999 | 1.000 | 0.983 | 0.967 | 0.988 | 0.998 | 0.995 | 0.876 | 0.441 | 0.524 | 0.609 | 0.513 | 0.655 | 0.806 | 0.780 | 0.842 | 0.744 | 0.778 | 0.829 | 0.682 | 0.684 | 0.697 | 0.615 | 0.944 |
| J1 | 0.449 | 0.979 | 0.983 | 1.000 | 0.997 | 0.950 | 0.992 | 0.980 | 0.789 | 0.279 | 0.370 | 0.464 | 0.358 | 0.507 | 0.693 | 0.661 | 0.742 | 0.649 | 0.740 | 0.777 | 0.622 | 0.638 | 0.616 | 0.542 | 0.876 |
| J2 | 0.386 | 0.962 | 0.967 | 0.997 | 1.000 | 0.926 | 0.980 | 0.965 | 0.748 | 0.211 | 0.304 | 0.400 | 0.291 | 0.443 | 0.642 | 0.609 | 0.696 | 0.608 | 0.720 | 0.751 | 0.595 | 0.617 | 0.581 | 0.510 | 0.842 |
| J3 | 0.704 | 0.985 | 0.988 | 0.950 | 0.926 | 1.000 | 0.980 | 0.992 | 0.940 | 0.501 | 0.577 | 0.655 | 0.568 | 0.720 | 0.883 | 0.862 | 0.914 | 0.839 | 0.849 | 0.898 | 0.777 | 0.772 | 0.796 | 0.722 | 0.983 |
| J4 | 0.555 | 0.996 | 0.998 | 0.992 | 0.980 | 0.980 | 1.000 | 0.995 | 0.854 | 0.388 | 0.474 | 0.561 | 0.462 | 0.608 | 0.775 | 0.747 | 0.816 | 0.721 | 0.775 | 0.820 | 0.672 | 0.679 | 0.680 | 0.601 | 0.928 |
| J5 | 0.613 | 0.991 | 0.995 | 0.980 | 0.965 | 0.992 | 0.995 | 1.000 | 0.896 | 0.403 | 0.486 | 0.572 | 0.476 | 0.635 | 0.820 | 0.795 | 0.861 | 0.785 | 0.833 | 0.874 | 0.743 | 0.748 | 0.750 | 0.679 | 0.954 |
| J6 | 0.892 | 0.873 | 0.876 | 0.789 | 0.748 | 0.940 | 0.854 | 0.896 | 1.000 | 0.640 | 0.694 | 0.747 | 0.688 | 0.839 | 0.985 | 0.978 | 0.997 | 0.965 | 0.903 | 0.950 | 0.889 | 0.865 | 0.926 | 0.868 | 0.986 |
| P1 | 0.845 | 0.466 | 0.441 | 0.279 | 0.211 | 0.501 | 0.388 | 0.403 | 0.640 | 1.000 | 0.995 | 0.980 | 0.997 | 0.955 | 0.751 | 0.760 | 0.688 | 0.556 | 0.274 | 0.384 | 0.319 | 0.245 | 0.439 | 0.346 | 0.607 |
| P2 | 0.857 | 0.548 | 0.524 | 0.370 | 0.304 | 0.577 | 0.474 | 0.486 | 0.694 | 0.995 | 1.000 | 0.995 | 1.000 | 0.973 | 0.791 | 0.797 | 0.735 | 0.596 | 0.332 | 0.442 | 0.363 | 0.293 | 0.479 | 0.380 | 0.672 |
| P3 | 0.865 | 0.631 | 0.609 | 0.464 | 0.400 | 0.655 | 0.561 | 0.572 | 0.747 | 0.980 | 0.995 | 1.000 | 0.993 | 0.983 | 0.829 | 0.832 | 0.781 | 0.638 | 0.396 | 0.506 | 0.413 | 0.347 | 0.522 | 0.419 | 0.737 |
| P4 | 0.858 | 0.537 | 0.513 | 0.358 | 0.291 | 0.568 | 0.462 | 0.476 | 0.688 | 0.997 | 1.000 | 0.993 | 1.000 | 0.972 | 0.788 | 0.794 | 0.731 | 0.594 | 0.327 | 0.437 | 0.361 | 0.290 | 0.477 | 0.379 | 0.664 |
| P5 | 0.940 | 0.671 | 0.655 | 0.507 | 0.443 | 0.720 | 0.608 | 0.635 | 0.839 | 0.955 | 0.973 | 0.983 | 0.972 | 1.000 | 0.911 | 0.915 | 0.871 | 0.764 | 0.541 | 0.637 | 0.567 | 0.505 | 0.666 | 0.576 | 0.812 |
| W1 | 0.954 | 0.807 | 0.806 | 0.693 | 0.642 | 0.883 | 0.775 | 0.820 | 0.985 | 0.751 | 0.791 | 0.829 | 0.788 | 0.911 | 1.000 | 0.999 | 0.995 | 0.956 | 0.840 | 0.898 | 0.848 | 0.811 | 0.904 | 0.842 | 0.954 |
| W2 | 0.966 | 0.781 | 0.780 | 0.661 | 0.609 | 0.862 | 0.747 | 0.795 | 0.978 | 0.760 | 0.797 | 0.832 | 0.794 | 0.915 | 0.999 | 1.000 | 0.992 | 0.958 | 0.833 | 0.891 | 0.849 | 0.810 | 0.907 | 0.848 | 0.941 |
| W3 | 0.925 | 0.840 | 0.842 | 0.742 | 0.696 | 0.914 | 0.816 | 0.861 | 0.997 | 0.688 | 0.735 | 0.781 | 0.731 | 0.871 | 0.995 | 0.992 | 1.000 | 0.968 | 0.883 | 0.933 | 0.880 | 0.850 | 0.925 | 0.867 | 0.973 |
| W4 | 0.902 | 0.733 | 0.744 | 0.649 | 0.608 | 0.839 | 0.721 | 0.785 | 0.965 | 0.556 | 0.596 | 0.638 | 0.594 | 0.764 | 0.956 | 0.958 | 0.968 | 1.000 | 0.945 | 0.967 | 0.965 | 0.942 | 0.990 | 0.963 | 0.912 |
| Y1 | 0.710 | 0.758 | 0.778 | 0.740 | 0.720 | 0.849 | 0.775 | 0.833 | 0.903 | 0.274 | 0.332 | 0.396 | 0.327 | 0.541 | 0.840 | 0.833 | 0.883 | 0.945 | 1.000 | 0.992 | 0.985 | 0.990 | 0.969 | 0.961 | 0.875 |
| Y2 | 0.773 | 0.813 | 0.829 | 0.777 | 0.751 | 0.898 | 0.820 | 0.874 | 0.950 | 0.384 | 0.442 | 0.506 | 0.437 | 0.637 | 0.898 | 0.891 | 0.933 | 0.967 | 0.992 | 1.000 | 0.975 | 0.972 | 0.973 | 0.950 | 0.927 |
| Y3 | 0.767 | 0.662 | 0.682 | 0.622 | 0.595 | 0.777 | 0.672 | 0.743 | 0.889 | 0.319 | 0.363 | 0.413 | 0.361 | 0.567 | 0.848 | 0.849 | 0.880 | 0.965 | 0.985 | 0.975 | 1.000 | 0.996 | 0.992 | 0.994 | 0.832 |
| Y4 | 0.712 | 0.661 | 0.684 | 0.638 | 0.617 | 0.772 | 0.679 | 0.748 | 0.865 | 0.245 | 0.293 | 0.347 | 0.290 | 0.505 | 0.811 | 0.810 | 0.850 | 0.942 | 0.990 | 0.972 | 0.996 | 1.000 | 0.978 | 0.985 | 0.815 |
| Y5 | 0.844 | 0.681 | 0.697 | 0.616 | 0.581 | 0.796 | 0.680 | 0.750 | 0.926 | 0.439 | 0.479 | 0.522 | 0.477 | 0.666 | 0.904 | 0.907 | 0.925 | 0.990 | 0.969 | 0.973 | 0.992 | 0.978 | 1.000 | 0.991 | 0.866 |
| Y6 | 0.792 | 0.595 | 0.615 | 0.542 | 0.510 | 0.722 | 0.601 | 0.679 | 0.868 | 0.346 | 0.380 | 0.419 | 0.379 | 0.576 | 0.842 | 0.848 | 0.867 | 0.963 | 0.961 | 0.950 | 0.994 | 0.985 | 0.991 | 1.000 | 0.796 |
| R | 0.822 | 0.942 | 0.944 | 0.876 | 0.842 | 0.983 | 0.928 | 0.954 | 0.986 | 0.607 | 0.672 | 0.737 | 0.664 | 0.812 | 0.954 | 0.941 | 0.973 | 0.912 | 0.875 | 0.927 | 0.832 | 0.815 | 0.866 | 0.796 | 1.000 |
