# Supplementary material for: Analysis of genetic and chemical variability of five Curcuma species based on DNA barcoding and HPLC fingerprints
Source: Front Plant Sci. 2023 Sep 6;14:1229041. doi: 10.3389/fpls.2023.1229041 (PMC10511903; doi:10.3389/fpls.2023.1229041)
Supplement: Supplementary file 3 [file Table_2.docx]

Table S2 The Euclidean distance of five *Curcuma* species based on standardized HPLC 17 characteristic peak areas

| Species | *C.* *wenyujin* | *C.* *aromatica* | *C.* *phaeocaulis* | *C.* *kwangsiensis* | *C.* *longa* |
| --- | --- | --- | --- | --- | --- |
| *C.* *wenyujin* | 0 |  |  |  |  |
| *C.* *aromatica* | 3.373 | 0 |  |  |  |
| *C.* *phaeocaulis* | 5.322 | 5.752 | 0 |  |  |
| *C.* *kwangsiensis* | 5.361 | 5.960 | 5.209 | 0 |  |
| *C.* *longa* | 6.239 | 6.392 | 6.861 | 6.998 | 0 |
