# Supplementary material for: Analysis of genetic and chemical variability of five Curcuma species based on DNA barcoding and HPLC fingerprints
Source: Front Plant Sci. 2023 Sep 6;14:1229041. doi: 10.3389/fpls.2023.1229041 (PMC10511903; doi:10.3389/fpls.2023.1229041)
Supplement: Supplementary file 4 [file Table_3.docx]

Table S3 The content of six compounds in different samples（n=3）

| Sample ID | Curdione mg/g | Furanodienon mg/g | Curcumenol mg/g | Germacrone mg/g | Curzerene mg/g | bate-elemene mg/g |
| --- | --- | --- | --- | --- | --- | --- |
| P1 | 0.6922±0.0314 | 0.8945±0.0239 | 0.5969±0.0112 | 0.5439±0.0034 | 0.1439±0.0021 | 2.5408±0.0312 |
| P2 | 0.9464±0.1298 | 1.7116±0.0288 | 0.0616±0.0065 | 1.4849±0.0218 | 0.5459±1.0021 | 1.3237±1.0312 |
| P3 | 0.6530±0.0029 | 1.4243±0.0122 | 0.0476±0.0000 | 1.5393±0.0374 | 0.2662±0.0021 | 0.8822±0.0312 |
| P4 | 1.0712±0.2519 | 1.9348±0.1014 | 1.2753±0.0617 | 1.5408±0.0206 | 0.7115±2.0021 | 1.7285±2.0312 |
| P5 | 0.9767±0.0024 | 1.3983±0.0054 | 0.6582±0.0001 | 0.5210±0.0146 | 0.1524±0.0021 | 0.8678±0.0312 |
| Y1 | 10.8201±0.0077 | 0.0803±0.0043 | 1.0091±0.0017 | 2.9545±0.0691 | 0.1955±0.0021 | 2.2918±0.0312 |
| Y2 | 9.3721±0.1242 | 0.1468±0.0041 | 1.0438±0.0104 | 2.8331±0.0025 | 0.2386±1.0021 | 1.5612±1.0312 |
| Y3 | 11.7542±0.1424 | 0.1148±0.0033 | 1.1672±0.0968 | 2.2652±0.0142 | 0.2290±1.0021 | 2.0115±1.0312 |
| Y4 | 12.1390±0.0581 | 0.0507±0.0004 | 1.4219±0.1241 | 2.4205±0.0413 | 0.4393±0.0021 | 3.0035±0.0312 |
| Y5 | 9.5606±0.0923 | 0.2362±0.0005 | 1.4891±0.0143 | 1.8712±0.0091 | 0.5325±0.0021 | 2.4650±0.0312 |
| Y6 | 6.8979±0.0367 | 0.1568±0.0174 | 0.3713±0.0020 | 1.0700±0.0160 | 0.2131±0.0021 | 1.2215±0.0312 |
| J1 | 0.7532±0.0013 | 0.0235±0.0020 | 0.0689±0.0020 | 2.4543±0.1028 | 0.2664±0.0021 | 5.1776±0.0312 |
| J2 | 1.0010±0.0139 | 0.0494±0.0005 | 0.3032±0.0007 | 22.1229±0.0492 | 0.3395±0.0021 | 3.5247±0.0312 |
| J3 | 0.8948±0.0083 | 0.0249±0.0003 | 0.1839±0.0072 | 1.1907±0.1273 | 0.2701±0.0021 | 3.6267±0.0312 |
| J4 | 0.9269±0.0334 | 0.0743±0.0007 | 0.1334±0.0026 | 2.5967±0.0450 | 0.3952±0.0021 | 4.4267±0.0312 |
| J5 | 1.7581±0.0306 | 0.0553±0.0010 | 0.1893±0.0064 | 2.4294±0.0389 | 0.4084±0.0021 | 2.4254±0.0312 |
| J6 | 3.8663±0.1959 | 0.2393±0.0052 | 0.4666±0.0050 | 1.6105±0.0246 | 0.6186±1.0021 | 4.9487±1.0312 |
| W1 | 10.2745±0.0450 | 0.2271±0.0012 | 0.7674±0.0035 | 3.5317±0.0302 | 1.0583±0.0021 | 3.0978±0.0312 |
| W2 | 8.4492±0.0173 | 0.0987±0.0034 | 0.8171±0.0004 | 2.6226±0.0238 | 1.0326±0.0021 | 2.5918±0.0312 |
| W3 | 8.9062±0.1122 | 0.1826±0.0924 | 0.8229±0.2484 | 3.2340±0.0321 | 1.6192±1.0021 | 2.9178±1.0312 |
| W4 | 9.4689±0.6222 | 0.5586±0.0078 | 1.1011±0.0530 | 2.1019±0.0825 | 0.7883±6.0021 | 1.9663±6.0312 |
| G1 | 2.7468±0.1212 | 3.2355±0.0219 | 0.2846±0.0125 | 0.5372±0.0162 | 0.2100±1.0021 | 0.8571±1.0312 |
| G2 | 1.5848±0.1419 | 10.8051±0.0590 | 0.5280±0.0574 | 5.3398±0.3858 | 0.3627±0.1104 | 1.1523±0.0403 |
| G3 | 1.1808±0.0348 | 8.9100±0.3125 | 0.5946±0.6096 | 2.9846±0.1017 | 0.1215±0.0015 | 0.8728±0.0101 |
