# Supplementary material for: Analysis of genetic and chemical variability of five Curcuma species based on DNA barcoding and HPLC fingerprints
Source: Front Plant Sci. 2023 Sep 6;14:1229041. doi: 10.3389/fpls.2023.1229041 (PMC10511903; doi:10.3389/fpls.2023.1229041)
Supplement: Supplementary file 5 [file Table_4.docx]

Table S4 Matrix A data list

| Sample | P1 | P2 | P3 | P4 | P5 | Y1 | Y2 | Y3 | Y4 | Y5 | Y6 | J1 | J2 | J3 | J4 | J5 | J6 | W1 | W2 | W3 | W4 | G1 | G2 | G3 |
| --- | --- | --- | --- | --- | --- | --- | --- | --- | --- | --- | --- | --- | --- | --- | --- | --- | --- | --- | --- | --- | --- | --- | --- | --- |
| P1 | 0.0000 | 0.0161 | 0.0168 | 0.0101 | 0.0101 | 0.0255 | 0.0235 | 0.0271 | 0.0249 | 0.0260 | 0.0121 | 0.0121 | 0.0128 | 0.0128 | 0.0068 | 0.0128 | 0.0114 | 0.0161 | 0.0167 | 0.0141 | 0.0265 | 0.0182 | 0.0209 | 0.0209 |
| P2 | 0.0161 | 0.0000 | 0.0115 | 0.0100 | 0.0097 | 0.0273 | 0.0299 | 0.0347 | 0.0247 | 0.0290 | 0.0105 | 0.0112 | 0.0118 | 0.0112 | 0.0154 | 0.0118 | 0.0123 | 0.0169 | 0.0170 | 0.0144 | 0.0348 | 0.0197 | 0.0230 | 0.0230 |
| P3 | 0.0168 | 0.0115 | 0.0000 | 0.0120 | 0.0084 | 0.0273 | 0.0273 | 0.0347 | 0.0286 | 0.0290 | 0.0125 | 0.0118 | 0.0125 | 0.0125 | 0.0168 | 0.0144 | 0.0156 | 0.0182 | 0.0170 | 0.0151 | 0.0355 | 0.0197 | 0.0230 | 0.0230 |
| P4 | 0.0101 | 0.0100 | 0.0120 | 0.0000 | 0.0101 | 0.0282 | 0.0275 | 0.0238 | 0.0275 | 0.0246 | 0.0121 | 0.0121 | 0.0148 | 0.0148 | 0.0095 | 0.0141 | 0.0134 | 0.0161 | 0.0174 | 0.0154 | 0.0251 | 0.0222 | 0.0242 | 0.0242 |
| P5 | 0.0101 | 0.0097 | 0.0084 | 0.0101 | 0.0000 | 0.0242 | 0.0209 | 0.0315 | 0.0229 | 0.0263 | 0.0059 | 0.0053 | 0.0060 | 0.0066 | 0.0108 | 0.0073 | 0.0079 | 0.0105 | 0.0086 | 0.0059 | 0.0329 | 0.0126 | 0.0159 | 0.0159 |
| Y1 | 0.0255 | 0.0273 | 0.0273 | 0.0282 | 0.0242 | 0.0000 | 0.0168 | 0.0179 | 0.0084 | 0.0141 | 0.0217 | 0.0197 | 0.0205 | 0.0224 | 0.0256 | 0.0191 | 0.0197 | 0.0267 | 0.0263 | 0.0211 | 0.0208 | 0.0283 | 0.0323 | 0.0323 |
| Y2 | 0.0235 | 0.0299 | 0.0273 | 0.0275 | 0.0209 | 0.0168 | 0.0000 | 0.0199 | 0.0123 | 0.0141 | 0.0165 | 0.0171 | 0.0185 | 0.0171 | 0.0222 | 0.0224 | 0.0191 | 0.0241 | 0.0210 | 0.0211 | 0.0221 | 0.0230 | 0.0270 | 0.0270 |
| Y3 | 0.0271 | 0.0347 | 0.0347 | 0.0238 | 0.0315 | 0.0179 | 0.0199 | 0.0000 | 0.0219 | 0.0174 | 0.0301 | 0.0321 | 0.0322 | 0.0328 | 0.0225 | 0.0328 | 0.0314 | 0.0341 | 0.0354 | 0.0348 | 0.0188 | 0.0368 | 0.0409 | 0.0409 |
| Y4 | 0.0249 | 0.0247 | 0.0286 | 0.0275 | 0.0229 | 0.0084 | 0.0123 | 0.0219 | 0.0000 | 0.0121 | 0.0204 | 0.0184 | 0.0198 | 0.0184 | 0.0223 | 0.0184 | 0.0197 | 0.0234 | 0.0250 | 0.0198 | 0.0174 | 0.0277 | 0.0310 | 0.0310 |
| Y5 | 0.0260 | 0.0290 | 0.0290 | 0.0246 | 0.0263 | 0.0141 | 0.0141 | 0.0174 | 0.0121 | 0.0000 | 0.0264 | 0.0264 | 0.0264 | 0.0264 | 0.0241 | 0.0264 | 0.0277 | 0.0263 | 0.0277 | 0.0263 | 0.0182 | 0.0331 | 0.0365 | 0.0365 |
| Y6 | 0.0121 | 0.0105 | 0.0125 | 0.0121 | 0.0059 | 0.0217 | 0.0165 | 0.0301 | 0.0204 | 0.0264 | 0.0000 | 0.0059 | 0.0079 | 0.0046 | 0.0087 | 0.0079 | 0.0066 | 0.0112 | 0.0131 | 0.0105 | 0.0349 | 0.0112 | 0.0125 | 0.0125 |
| J1 | 0.0121 | 0.0112 | 0.0118 | 0.0121 | 0.0053 | 0.0197 | 0.0171 | 0.0321 | 0.0184 | 0.0264 | 0.0059 | 0.0000 | 0.0066 | 0.0059 | 0.0067 | 0.0066 | 0.0066 | 0.0092 | 0.0112 | 0.0059 | 0.0330 | 0.0125 | 0.0145 | 0.0145 |
| J2 | 0.0128 | 0.0118 | 0.0125 | 0.0148 | 0.0060 | 0.0205 | 0.0185 | 0.0322 | 0.0198 | 0.0264 | 0.0079 | 0.0066 | 0.0000 | 0.0079 | 0.0087 | 0.0085 | 0.0053 | 0.0099 | 0.0099 | 0.0092 | 0.0303 | 0.0112 | 0.0165 | 0.0165 |
| J3 | 0.0128 | 0.0112 | 0.0125 | 0.0148 | 0.0066 | 0.0224 | 0.0171 | 0.0328 | 0.0184 | 0.0264 | 0.0046 | 0.0059 | 0.0079 | 0.0000 | 0.0087 | 0.0085 | 0.0098 | 0.0105 | 0.0118 | 0.0099 | 0.0336 | 0.0125 | 0.0145 | 0.0145 |
| J4 | 0.0068 | 0.0154 | 0.0168 | 0.0095 | 0.0108 | 0.0256 | 0.0222 | 0.0225 | 0.0223 | 0.0241 | 0.0087 | 0.0067 | 0.0087 | 0.0087 | 0.0000 | 0.0094 | 0.0101 | 0.0148 | 0.0154 | 0.0121 | 0.0219 | 0.0162 | 0.0169 | 0.0169 |
| J5 | 0.0128 | 0.0118 | 0.0144 | 0.0141 | 0.0073 | 0.0191 | 0.0224 | 0.0328 | 0.0184 | 0.0264 | 0.0079 | 0.0066 | 0.0085 | 0.0085 | 0.0094 | 0.0000 | 0.0085 | 0.0138 | 0.0151 | 0.0072 | 0.0329 | 0.0171 | 0.0178 | 0.0178 |
| J6 | 0.0114 | 0.0123 | 0.0156 | 0.0134 | 0.0079 | 0.0197 | 0.0191 | 0.0314 | 0.0197 | 0.0277 | 0.0066 | 0.0066 | 0.0053 | 0.0098 | 0.0101 | 0.0085 | 0.0000 | 0.0085 | 0.0111 | 0.0072 | 0.0335 | 0.0145 | 0.0178 | 0.0178 |
| W1 | 0.0161 | 0.0169 | 0.0182 | 0.0161 | 0.0105 | 0.0267 | 0.0241 | 0.0341 | 0.0234 | 0.0263 | 0.0112 | 0.0092 | 0.0099 | 0.0105 | 0.0148 | 0.0138 | 0.0085 | 0.0000 | 0.0026 | 0.0079 | 0.0322 | 0.0132 | 0.0171 | 0.0171 |
| W2 | 0.0167 | 0.0170 | 0.0170 | 0.0174 | 0.0086 | 0.0263 | 0.0210 | 0.0354 | 0.0250 | 0.0277 | 0.0131 | 0.0112 | 0.0099 | 0.0118 | 0.0154 | 0.0151 | 0.0111 | 0.0026 | 0.0000 | 0.0111 | 0.0329 | 0.0151 | 0.0191 | 0.0191 |
| W3 | 0.0141 | 0.0144 | 0.0151 | 0.0154 | 0.0059 | 0.0211 | 0.0211 | 0.0348 | 0.0198 | 0.0263 | 0.0105 | 0.0059 | 0.0092 | 0.0099 | 0.0121 | 0.0072 | 0.0072 | 0.0079 | 0.0111 | 0.0000 | 0.0329 | 0.0171 | 0.0185 | 0.0185 |
| W4 | 0.0265 | 0.0348 | 0.0355 | 0.0251 | 0.0329 | 0.0208 | 0.0221 | 0.0188 | 0.0174 | 0.0182 | 0.0349 | 0.0330 | 0.0303 | 0.0336 | 0.0219 | 0.0329 | 0.0335 | 0.0322 | 0.0329 | 0.0329 | 0.0000 | 0.0398 | 0.0438 | 0.0438 |
| G1 | 0.0182 | 0.0197 | 0.0197 | 0.0222 | 0.0126 | 0.0283 | 0.0230 | 0.0368 | 0.0277 | 0.0331 | 0.0112 | 0.0125 | 0.0112 | 0.0125 | 0.0162 | 0.0171 | 0.0145 | 0.0132 | 0.0151 | 0.0171 | 0.0398 | 0.0000 | 0.0052 | 0.0052 |
| G2 | 0.0209 | 0.0230 | 0.0230 | 0.0242 | 0.0159 | 0.0323 | 0.0270 | 0.0409 | 0.0310 | 0.0365 | 0.0125 | 0.0145 | 0.0165 | 0.0145 | 0.0169 | 0.0178 | 0.0178 | 0.0171 | 0.0191 | 0.0185 | 0.0438 | 0.0052 | 0.0000 | 0.0000 |
| G3 | 0.0209 | 0.0230 | 0.0230 | 0.0242 | 0.0159 | 0.0323 | 0.0270 | 0.0409 | 0.0310 | 0.0365 | 0.0125 | 0.0145 | 0.0165 | 0.0145 | 0.0169 | 0.0178 | 0.0178 | 0.0171 | 0.0191 | 0.0185 | 0.0438 | 0.0052 | 0.0000 | 0.0000 |
