# Supplementary material for: Analysis of genetic and chemical variability of five Curcuma species based on DNA barcoding and HPLC fingerprints
Source: Front Plant Sci. 2023 Sep 6;14:1229041. doi: 10.3389/fpls.2023.1229041 (PMC10511903; doi:10.3389/fpls.2023.1229041)
Supplement: Supplementary file 6 [file Table_5.docx]

Table S5 Matrix B data list

| Sample | P1 | P2 | P3 | P4 | P5 | Y1 | Y2 | Y3 | Y4 | Y5 | Y6 | J1 | J2 | J3 | J4 | J5 | J6 | W1 | W2 | W3 | W4 | G1 | G2 | G3 |
| --- | --- | --- | --- | --- | --- | --- | --- | --- | --- | --- | --- | --- | --- | --- | --- | --- | --- | --- | --- | --- | --- | --- | --- | --- |
| P1 | 0.0000 | 3.4739 | 2.5097 | 4.2461 | 2.2866 | 5.5608 | 5.0435 | 5.7064 | 6.6222 | 5.5157 | 3.9573 | 4.3061 | 9.3460 | 4.1949 | 4.5131 | 6.8291 | 6.7771 | 6.1335 | 5.2071 | 6.8571 | 5.0088 | 5.0235 | 6.5853 | 5.8238 |
| P2 | 3.4739 | 0.0000 | 1.9652 | 2.9359 | 3.6681 | 6.2651 | 5.9216 | 6.5042 | 7.2770 | 6.0146 | 4.7241 | 5.2974 | 9.9687 | 5.4799 | 5.9984 | 6.9020 | 6.9625 | 5.5440 | 4.6498 | 6.0195 | 5.3837 | 4.6421 | 5.8913 | 5.8244 |
| P3 | 2.5097 | 1.9652 | 0.0000 | 3.9454 | 2.4489 | 6.2269 | 5.8620 | 6.4290 | 7.4956 | 6.2505 | 4.1526 | 4.9467 | 9.6662 | 5.3701 | 5.9876 | 6.7726 | 6.8343 | 6.2938 | 5.3217 | 6.8738 | 5.4893 | 4.6043 | 6.2796 | 5.5803 |
| P4 | 4.2461 | 2.9359 | 3.9454 | 0.0000 | 4.5255 | 6.4554 | 6.0373 | 6.5242 | 6.9795 | 5.5433 | 5.8043 | 6.4297 | 10.5368 | 6.3471 | 5.8071 | 7.6709 | 7.5063 | 5.4972 | 4.6430 | 5.8101 | 5.2208 | 5.7026 | 6.4579 | 6.4255 |
| P5 | 2.2866 | 3.6681 | 2.4489 | 4.5255 | 0.0000 | 5.3873 | 5.0792 | 5.6133 | 6.6898 | 5.3795 | 3.1679 | 4.5867 | 9.4921 | 5.0634 | 5.1182 | 6.4915 | 6.4242 | 6.3400 | 5.1930 | 6.8598 | 4.7563 | 4.0692 | 5.8132 | 4.8845 |
| Y1 | 5.5608 | 6.2651 | 6.2269 | 6.4554 | 5.3873 | 0.0000 | 0.9831 | 0.7346 | 1.8198 | 1.7170 | 3.1597 | 5.4892 | 9.0592 | 5.4435 | 4.9250 | 6.7873 | 6.6538 | 3.8447 | 3.5785 | 5.3571 | 3.5155 | 5.5065 | 6.4984 | 6.6844 |
| Y2 | 5.0435 | 5.9216 | 5.8620 | 6.0373 | 5.0792 | 0.9831 | 0.0000 | 1.1042 | 1.9299 | 1.7244 | 3.0722 | 4.8761 | 8.4749 | 4.7278 | 4.1905 | 6.4258 | 6.2774 | 3.6083 | 3.3233 | 5.1076 | 3.1129 | 5.4592 | 6.4968 | 6.6040 |
| Y3 | 5.7064 | 6.5042 | 6.4290 | 6.5242 | 5.6133 | 0.7346 | 1.1042 | 0.0000 | 1.6257 | 1.6377 | 3.5598 | 5.8204 | 9.2696 | 5.7271 | 5.1069 | 7.0019 | 6.8423 | 3.9761 | 3.7760 | 5.4516 | 3.6135 | 5.9795 | 6.9279 | 7.0111 |
| Y4 | 6.6222 | 7.2770 | 7.4956 | 6.9795 | 6.6898 | 1.8198 | 1.9299 | 1.6257 | 0.0000 | 1.7761 | 4.7070 | 6.2324 | 9.5147 | 5.9182 | 4.9968 | 7.5026 | 7.3081 | 3.8354 | 3.9386 | 5.1912 | 3.8706 | 6.7495 | 7.3594 | 7.7662 |
| Y5 | 5.5157 | 6.0146 | 6.2505 | 5.5433 | 5.3795 | 1.7170 | 1.7244 | 1.6377 | 1.7761 | 0.0000 | 3.8098 | 5.9235 | 9.5246 | 5.6832 | 4.7214 | 6.9219 | 6.6970 | 3.3403 | 2.9216 | 4.5035 | 3.0770 | 5.6342 | 6.4017 | 6.6597 |
| Y6 | 3.9573 | 4.7241 | 4.1526 | 5.8043 | 3.1679 | 3.1597 | 3.0722 | 3.5598 | 4.7070 | 3.8098 | 0.0000 | 4.2306 | 8.7297 | 4.6463 | 4.8085 | 5.4917 | 5.4813 | 4.5312 | 3.6853 | 5.6122 | 3.3319 | 3.6311 | 5.4005 | 5.1222 |
| J1 | 4.3061 | 5.2974 | 4.9467 | 6.4297 | 4.5867 | 5.4892 | 4.8761 | 5.8204 | 6.2324 | 5.9235 | 4.2306 | 0.0000 | 7.2454 | 1.7255 | 2.9567 | 5.8981 | 5.9688 | 5.9597 | 5.4777 | 6.7240 | 4.6891 | 5.6030 | 6.8368 | 6.8355 |
| J2 | 9.3460 | 9.9687 | 9.6662 | 10.5368 | 9.4921 | 9.0592 | 8.4749 | 9.2696 | 9.5147 | 9.5246 | 8.7297 | 7.2454 | 0.0000 | 7.3160 | 7.9133 | 7.4369 | 7.4413 | 9.3040 | 9.2676 | 9.8736 | 8.8194 | 9.2532 | 10.2264 | 10.1283 |
| J3 | 4.1949 | 5.4799 | 5.3701 | 6.3471 | 5.0634 | 5.4435 | 4.7278 | 5.7271 | 5.9182 | 5.6832 | 4.6463 | 1.7255 | 7.3160 | 0.0000 | 2.3881 | 6.0858 | 6.1169 | 5.5865 | 5.1955 | 6.2656 | 4.6981 | 5.8845 | 6.9642 | 7.0488 |
| J4 | 4.5131 | 5.9984 | 5.9876 | 5.8071 | 5.1182 | 4.9250 | 4.1905 | 5.1069 | 4.9968 | 4.7214 | 4.8085 | 2.9567 | 7.9133 | 2.3881 | 0.0000 | 6.4324 | 6.2540 | 5.2210 | 4.8140 | 5.9220 | 3.8562 | 6.1331 | 6.9783 | 7.0921 |
| J5 | 6.8291 | 6.9020 | 6.7726 | 7.6709 | 6.4915 | 6.7873 | 6.4258 | 7.0019 | 7.5026 | 6.9219 | 5.4917 | 5.8981 | 7.4369 | 6.0858 | 6.4324 | 0.0000 | 0.6045 | 6.6012 | 6.2159 | 6.9779 | 5.9158 | 6.4233 | 7.3624 | 7.4118 |
| J6 | 6.7771 | 6.9625 | 6.8343 | 7.5063 | 6.4242 | 6.6538 | 6.2774 | 6.8423 | 7.3081 | 6.6970 | 5.4813 | 5.9688 | 7.4413 | 6.1169 | 6.2540 | 0.6045 | 0.0000 | 6.5128 | 6.1114 | 6.8837 | 5.7410 | 6.4322 | 7.3339 | 7.3589 |
| W1 | 6.1335 | 5.5440 | 6.2938 | 5.4972 | 6.3400 | 3.8447 | 3.6083 | 3.9761 | 3.8354 | 3.3403 | 4.5312 | 5.9597 | 9.3040 | 5.5865 | 5.2210 | 6.6012 | 6.5128 | 0.0000 | 1.3639 | 2.3356 | 2.8018 | 5.4282 | 6.0913 | 6.7544 |
| W2 | 5.2071 | 4.6498 | 5.3217 | 4.6430 | 5.1930 | 3.5785 | 3.3233 | 3.7760 | 3.9386 | 2.9216 | 3.6853 | 5.4777 | 9.2676 | 5.1955 | 4.8140 | 6.2159 | 6.1114 | 1.3639 | 0.0000 | 2.3685 | 2.3422 | 4.5751 | 5.4969 | 5.9778 |
| W3 | 6.8571 | 6.0195 | 6.8738 | 5.8101 | 6.8598 | 5.3571 | 5.1076 | 5.4516 | 5.1912 | 4.5035 | 5.6122 | 6.7240 | 9.8736 | 6.2656 | 5.9220 | 6.9779 | 6.8837 | 2.3356 | 2.3685 | 0.0000 | 3.5741 | 5.8329 | 6.2807 | 6.9259 |
| W4 | 5.0088 | 5.3837 | 5.4893 | 5.2208 | 4.7563 | 3.5155 | 3.1129 | 3.6135 | 3.8706 | 3.0770 | 3.3319 | 4.6891 | 8.8194 | 4.6981 | 3.8562 | 5.9158 | 5.7410 | 2.8018 | 2.3422 | 3.5741 | 0.0000 | 4.7892 | 5.7599 | 5.8589 |
| G1 | 5.0235 | 4.6421 | 4.6043 | 5.7026 | 4.0692 | 5.5065 | 5.4592 | 5.9795 | 6.7495 | 5.6342 | 3.6311 | 5.6030 | 9.2532 | 5.8845 | 6.1331 | 6.4233 | 6.4322 | 5.4282 | 4.5751 | 5.8329 | 4.7892 | 0.0000 | 2.3536 | 2.2992 |
| G2 | 6.5853 | 5.8913 | 6.2796 | 6.4579 | 5.8132 | 6.4984 | 6.4968 | 6.9279 | 7.3594 | 6.4017 | 5.4005 | 6.8368 | 10.2264 | 6.9642 | 6.9783 | 7.3624 | 7.3339 | 6.0913 | 5.4969 | 6.2807 | 5.7599 | 2.3536 | 0.0000 | 2.2653 |
| G3 | 5.8238 | 5.8244 | 5.5803 | 6.4255 | 4.8845 | 6.6844 | 6.6040 | 7.0111 | 7.7662 | 6.6597 | 5.1222 | 6.8355 | 10.1283 | 7.0488 | 7.0921 | 7.4118 | 7.3589 | 6.7544 | 5.9778 | 6.9259 | 5.8589 | 2.2992 | 2.2653 | 0.0000 |
