# Supplementary material for: Analysis of genetic and chemical variability of five Curcuma species based on DNA barcoding and HPLC fingerprints
Source: Front Plant Sci. 2023 Sep 6;14:1229041. doi: 10.3389/fpls.2023.1229041 (PMC10511903; doi:10.3389/fpls.2023.1229041)
Supplement: Supplementary file 7 [file Table_6.docx]

Table S6 Matrix C data list

| Sample | P1 | P2 | P3 | P4 | P5 | Y1 | Y2 | Y3 | Y4 | Y5 | Y6 | J1 | J2 | J3 | J4 | J5 | J6 | W1 | W2 | W3 | W4 | G1 | G2 | G3 |
| --- | --- | --- | --- | --- | --- | --- | --- | --- | --- | --- | --- | --- | --- | --- | --- | --- | --- | --- | --- | --- | --- | --- | --- | --- |
| P1 | 0.000 | 1.920 | 1.840 | 2.306 | 1.331 | 2.566 | 2.426 | 2.896 | 3.348 | 3.052 | 1.848 | 2.461 | 5.250 | 1.335 | 2.010 | 1.298 | 2.454 | 3.474 | 3.120 | 4.588 | 2.965 | 1.588 | 4.524 | 2.673 |
| P2 | 1.920 | 0.000 | 0.857 | 2.759 | 1.773 | 3.368 | 3.084 | 3.660 | 4.212 | 3.862 | 1.835 | 3.171 | 5.270 | 2.041 | 2.535 | 1.130 | 3.095 | 3.368 | 2.972 | 4.117 | 3.153 | 1.281 | 4.230 | 2.687 |
| P3 | 1.840 | 0.857 | 0.000 | 3.077 | 1.418 | 3.383 | 3.060 | 3.681 | 4.403 | 4.075 | 1.664 | 3.406 | 5.353 | 2.215 | 2.852 | 1.408 | 3.562 | 3.944 | 3.551 | 4.842 | 3.524 | 0.835 | 4.295 | 2.506 |
| P4 | 2.306 | 2.759 | 3.077 | 0.000 | 2.199 | 2.811 | 2.432 | 2.845 | 2.888 | 2.184 | 2.853 | 4.052 | 5.642 | 3.150 | 3.473 | 2.683 | 3.216 | 2.863 | 2.333 | 3.437 | 2.012 | 2.774 | 4.463 | 3.048 |
| P5 | 1.331 | 1.773 | 1.418 | 2.199 | 0.000 | 2.713 | 2.272 | 2.906 | 3.624 | 3.183 | 1.570 | 3.695 | 5.624 | 2.469 | 3.166 | 1.863 | 3.570 | 3.814 | 3.347 | 4.805 | 2.960 | 0.993 | 4.264 | 2.195 |
| Y1 | 2.566 | 3.368 | 3.383 | 2.811 | 2.713 | 0.000 | 0.678 | 0.503 | 1.312 | 1.478 | 1.929 | 3.849 | 5.405 | 3.129 | 3.463 | 2.820 | 3.131 | 2.542 | 2.436 | 4.028 | 1.723 | 2.752 | 5.140 | 3.611 |
| Y2 | 2.426 | 3.084 | 3.060 | 2.432 | 2.272 | 0.678 | 0.000 | 0.716 | 1.646 | 1.485 | 1.676 | 4.080 | 5.452 | 3.190 | 3.614 | 2.703 | 3.392 | 2.662 | 2.411 | 4.011 | 1.578 | 2.395 | 4.938 | 3.297 |
| Y3 | 2.896 | 3.660 | 3.681 | 2.845 | 2.906 | 0.503 | 0.716 | 0.000 | 1.131 | 1.268 | 2.197 | 4.294 | 5.775 | 3.547 | 3.896 | 3.203 | 3.489 | 2.650 | 2.522 | 4.057 | 1.649 | 3.008 | 5.285 | 3.788 |
| Y4 | 3.348 | 4.212 | 4.403 | 2.888 | 3.624 | 1.312 | 1.646 | 1.131 | 0.000 | 0.795 | 3.057 | 4.350 | 5.889 | 3.831 | 3.999 | 3.649 | 3.269 | 2.309 | 2.310 | 3.618 | 1.584 | 3.798 | 5.653 | 4.391 |
| Y5 | 3.052 | 3.862 | 4.075 | 2.184 | 3.183 | 1.478 | 1.485 | 1.268 | 0.795 | 0.000 | 2.887 | 4.372 | 5.899 | 3.704 | 3.939 | 3.414 | 3.275 | 2.267 | 2.067 | 3.399 | 1.190 | 3.476 | 5.382 | 4.042 |
| Y6 | 1.848 | 1.835 | 1.664 | 2.853 | 1.570 | 1.929 | 1.676 | 2.197 | 3.057 | 2.887 | 0.000 | 3.493 | 5.490 | 2.375 | 2.976 | 1.681 | 3.222 | 3.064 | 2.753 | 4.300 | 2.438 | 1.014 | 4.703 | 2.893 |
| J1 | 2.461 | 3.171 | 3.406 | 4.052 | 3.695 | 3.849 | 4.080 | 4.294 | 4.350 | 4.372 | 3.493 | 0.000 | 4.873 | 1.281 | 0.707 | 2.227 | 1.522 | 3.831 | 3.807 | 4.852 | 4.208 | 3.493 | 5.594 | 4.395 |
| J2 | 5.250 | 5.270 | 5.353 | 5.642 | 5.624 | 5.405 | 5.452 | 5.775 | 5.889 | 5.899 | 5.490 | 4.873 | 0.000 | 4.975 | 4.701 | 4.762 | 5.105 | 5.389 | 5.458 | 6.122 | 5.698 | 5.557 | 6.259 | 5.654 |
| J3 | 1.335 | 2.041 | 2.215 | 3.150 | 2.469 | 3.129 | 3.190 | 3.547 | 3.831 | 3.704 | 2.375 | 1.281 | 4.975 | 0.000 | 0.800 | 1.079 | 1.697 | 3.392 | 3.193 | 4.462 | 3.437 | 2.241 | 5.009 | 3.488 |
| J4 | 2.010 | 2.535 | 2.852 | 3.473 | 3.166 | 3.463 | 3.614 | 3.896 | 3.999 | 3.939 | 2.976 | 0.707 | 4.701 | 0.800 | 0.000 | 1.590 | 1.267 | 3.323 | 3.233 | 4.315 | 3.657 | 2.943 | 5.233 | 3.959 |
| J5 | 1.298 | 1.130 | 1.408 | 2.683 | 1.863 | 2.820 | 2.703 | 3.203 | 3.649 | 3.414 | 1.681 | 2.227 | 4.762 | 1.079 | 1.590 | 0.000 | 2.219 | 3.002 | 2.700 | 4.014 | 2.909 | 1.457 | 4.653 | 3.037 |
| J6 | 2.454 | 3.095 | 3.562 | 3.216 | 3.570 | 3.131 | 3.392 | 3.489 | 3.269 | 3.275 | 3.222 | 1.522 | 5.105 | 1.697 | 1.267 | 2.219 | 0.000 | 2.527 | 2.551 | 3.515 | 3.059 | 3.452 | 5.441 | 4.322 |
| W1 | 3.474 | 3.368 | 3.944 | 2.863 | 3.814 | 2.542 | 2.662 | 2.650 | 2.309 | 2.267 | 3.064 | 3.831 | 5.389 | 3.392 | 3.323 | 3.002 | 2.527 | 0.000 | 0.629 | 1.600 | 1.436 | 3.638 | 5.440 | 4.493 |
| W2 | 3.120 | 2.972 | 3.551 | 2.333 | 3.347 | 2.436 | 2.411 | 2.522 | 2.310 | 2.067 | 2.753 | 3.807 | 5.458 | 3.193 | 3.233 | 2.700 | 2.551 | 0.629 | 0.000 | 1.658 | 1.089 | 3.226 | 5.242 | 4.160 |
| W3 | 4.588 | 4.117 | 4.842 | 3.437 | 4.805 | 4.028 | 4.011 | 4.057 | 3.618 | 3.399 | 4.300 | 4.852 | 6.122 | 4.462 | 4.315 | 4.014 | 3.515 | 1.600 | 1.658 | 0.000 | 2.522 | 4.662 | 6.067 | 5.403 |
| W4 | 2.965 | 3.153 | 3.524 | 2.012 | 2.960 | 1.723 | 1.578 | 1.649 | 1.584 | 1.190 | 2.438 | 4.208 | 5.698 | 3.437 | 3.657 | 2.909 | 3.059 | 1.436 | 1.089 | 2.522 | 0.000 | 3.020 | 5.059 | 3.803 |
| G1 | 1.588 | 1.281 | 0.835 | 2.774 | 0.993 | 2.752 | 2.395 | 3.008 | 3.798 | 3.476 | 1.014 | 3.493 | 5.557 | 2.241 | 2.943 | 1.457 | 3.452 | 3.638 | 3.226 | 4.662 | 3.020 | 0.000 | 4.586 | 2.643 |
| G2 | 4.524 | 4.230 | 4.295 | 4.463 | 4.264 | 5.140 | 4.938 | 5.285 | 5.653 | 5.382 | 4.703 | 5.594 | 6.259 | 5.009 | 5.233 | 4.653 | 5.441 | 5.440 | 5.242 | 6.067 | 5.059 | 4.586 | 0.000 | 2.146 |
| G3 | 2.673 | 2.687 | 2.506 | 3.048 | 2.195 | 3.611 | 3.297 | 3.788 | 4.391 | 4.042 | 2.893 | 4.395 | 5.654 | 3.488 | 3.959 | 3.037 | 4.322 | 4.493 | 4.160 | 5.403 | 3.803 | 2.643 | 2.146 | 0.000 |
