# Supplementary material for: Analysis of genetic and chemical variability of five Curcuma species based on DNA barcoding and HPLC fingerprints
Source: Front Plant Sci. 2023 Sep 6;14:1229041. doi: 10.3389/fpls.2023.1229041 (PMC10511903; doi:10.3389/fpls.2023.1229041)
Supplement: Supplementary file 8 [file Table_7.doc]

Table S7 The GenBank accession numbers of the new annotation sample

| Gene | Specimen | Species | GenBank accession |
| --- | --- | --- | --- |
| ITS2 | P1 | *C. phaecocaulis* | OR381155 |
|  | P2 | *C. phaecocaulis* | OR381156 |
|  | P3 | *C. phaecocaulis* | OR381157 |
|  | P4 | *C. phaecocaulis* | OR381158 |
|  | P5 | *C. phaecocaulis* | OR381159 |
|  | Y1 | C. aromatica | OR381164 |
|  | Y2 | C. aromatica | OR381165 |
|  | Y3 | C. aromatica | OR381166 |
|  | Y4 | C. aromatica | OR381167 |
|  | Y5 | C. aromatica | OR381168 |
|  | Y6 | C. aromatica | OR381169 |
|  | W1 | C. *wenyujin* | OR381160 |
|  | W2 | C. *wenyujin* | OR381161 |
|  | W3 | C. *wenyujin* | OR381162 |
|  | W4 | C. *wenyujin* | OR381163 |
|  | G1 | *C. kwangsiensis* | OR381146 |
|  | G2 | *C. kwangsiensis* | OR381147 |
|  | G3 | *C. kwangsiensis* | OR381148 |
|  | J1 | *C. longa* | OR381149 |
|  | J2 | *C. longa* | OR381150 |
|  | J3 | *C. longa* | OR381151 |
|  | J4 | *C. longa* | OR381152 |
|  | J5 | *C. longa* | OR381153 |
|  | J6 | *C. longa* | OR381154 |
| trnK | P1 | *C. phaecocaulis* | OR398148 |
|  | P2 | *C. phaecocaulis* | OR398149 |
|  | P3 | *C. phaecocaulis* | OR398150 |
|  | P4 | *C. phaecocaulis* | OR398151 |
|  | P5 | *C. phaecocaulis* | OR398152 |
|  | Y1 | C. aromatica | OR398157 |
|  | Y2 | C. aromatica | OR398158 |
|  | Y3 | C. aromatica | OR398159 |
|  | Y4 | C. aromatica | OR398160 |
|  | Y5 | C. aromatica | OR398161 |
|  | Y6 | C. aromatica | OR398162 |
|  | W1 | C. *wenyujin* | OR398153 |
|  | W2 | C. *wenyujin* | OR398154 |
|  | W3 | C. *wenyujin* | OR398155 |
|  | W4 | C. *wenyujin* | OR398156 |
|  | G1 | *C. kwangsiensis* | OR398163 |
|  | G2 | *C. kwangsiensis* | OR398164 |
|  | G3 | *C. kwangsiensis* | OR398165 |
|  | J1 | *C. longa* | OR398142 |
|  | J2 | *C. longa* | OR398143 |
|  | J3 | *C. longa* | OR398144 |
|  | J4 | *C. longa* | OR398145 |
|  | J5 | *C. longa* | OR398146 |
|  | J6 | *C. longa* | OR398147 |
